# Single-Cell Transcriptomes Reveal Characteristic Features of Mouse Hepatocytes with Liver Cholestatic Injury

**DOI:** 10.3390/cells8091069

**Published:** 2019-09-11

**Authors:** Na Chang, Lei Tian, Xiaofang Ji, Xuan Zhou, Lei Hou, Xinhao Zhao, Yuanru Yang, Lin Yang, Liying Li

**Affiliations:** Department of Cell Biology, Municipal Laboratory for Liver Protection and Regulation of Regeneration, Capital Medical University, Beijing 100069, China

**Keywords:** single-cell RNA sequencing, cholestatic liver injury, hepatocyte heterogeneity, inflammation, liver tissue repair

## Abstract

Hepatocytes are the main parenchymal cells of the liver and play important roles in liver homeostasis and disease process. The heterogeneity of normal hepatocytes has been reported, but there is little knowledge about hepatocyte subtype and distinctive functions during liver cholestatic injury. Bile duct ligation (BDL)-induced mouse liver injury model was employed, and single-cell RNA sequencing was performed. Western blot and qPCR were used to study gene expression. Immunofluoresence was employed to detect the expressions of marker genes in hepatocytes. We detected a specific hepatocyte cluster (BDL-6) expressing extracellular matrix genes, indicating these hepatocytes might undergo epithelia-mesenchymal transition. Hepatocytes of BDL-6 also performed tissue repair functions (such as angiogenesis) during cholestatic injury. We also found that four clusters of cholestatic hepatocytes (BDL-2, BDL-3, BDL-4, and BDL-5) were involved in inflammatory process in different ways. To be specific, BDL-2/3/5 were inflammation-regulated hepatocytes, while BDL-4 played a role in cell chemotaxis. Among these four clusters, BDL-5 was special. because the hepatocytes of BDL-5 were proliferating hepatocytes. Our analysis provided more knowledge of hepatocyte distinctive functions in injured liver and gave rise to future treatment aiming at hepatocytes.

## 1. Introduction

Cholestatic liver injury is a common clinic symptom that is characterized by impaired bile flow in the liver. There are various reasons for cholestasis, such as acute hepatitis, viral infection, alcoholic liver disease, and drug-induced liver injury. In the liver, the accumulation of highly toxic bile acids in the hepatocytes leads to cytotoxicity and causes the death of hepatocytes. If left untreated, cholestasis will cause liver fibrosis, cirrhosis, and liver failure [1,2]. Inflammation is a character of cholestasis and anti-inflammation has been considered as a therapeutic target of cholestasis [3]. For this reason, the role of hepatic non-parenchymal cells (NPCs), especially immune cells (such as neutrophils and macrophages), has been well studied in cholestatic liver injury [1,3]. However, little research has been done on hepatocyte function during cholestasis.

As the main component of liver, hepatocytes make up ~80% of liver cells and are also important players in liver injury [4,5,6]. It has been reported that hepatocytes release cytokines and inflammatory extracellular vesicles to mediate liver inflammation [7,8,9]. In a recent study, hepatocyte-specific suppression of microRNA mitigates liver fibrosis [10]. On the other hand, hepatocytes are also involved in liver injury by participating in liver repair process. During liver injury, the reparation includes angiogenesis, extracellular matrix (ECM) component alteration and ECM reorganization. If the repair process is out of control, collagen will accumulate abnormally in the liver tissue. Then, liver fibrosis will occur. It has been reported that hepatocytes undergo epithelial-mesenchymal transition (EMT) during liver fibrosis [11]. EMT is a pathological process characterized by loss of epithelial features (such as low-expression of E-cadherin) and high expression of mesenchymal cell-related genes (such as Nestin and Cx43). The EMT of hepatocytes has been well studied in hepatocellular carcinoma [12]. However, whether EMT occurs in the process of liver fibrosis remains a controversial issue. Some studies have shown that hepatocytes play an important role in tissue repair and fibrogenesis through EMT. In this way, hepatocytes are transformed into cells that produce ECM and produce collagen to participate in tissue repair or fibrogenesis [11,13]. However, some researchers also reported that EMT did not occur in mouse liver fibrosis because no ECM-producing hepatocytes were found in lineage-tracking mice [14]. Therefore, it is worthwhile to study whether hepatocytes undergo EMT during cholestatic liver injury.

Single-cell RNA sequencing (scRNA-seq) could reveal the transcriptional heterogeneity of complex tissues or cells [15,16,17]. Recent research has identified different clusters of normal hepatocytes [18,19,20,21]. However, the knowledge of injured hepatocyte variation is limited. Therefore, figuring out hepatocyte changes after cholestatic liver injury will provide new view in cholestasis-injured liver treatment.

In this work, we aimed at exploring the cellular heterogeneity and characterizing the transcriptomic profile of cholestatic hepatocytes at the single-cell level. Bile duct ligation (BDL) was preformed to induce mouse cholestatic liver injury. scRNA-seq (10× Genomics) was used to identify expression profile of cells isolated from injured liver. We identified six clusters of hepatocytes isolated from injured liver. Among these cholestatic hepatocytes, we identified hepatocytes involved in tissue repair and liver inflammation. Furthermore, the repair-related hepatocytes underwent EMT during cholestasis-induced liver injury. The analysis revealed different functions of hepatocyte subtypes and their changes after liver injury.

## 2. Materials and Methods

### 2.1. Materials

LPS and Collagenase IV were obtained from Sigma (St. Louis, MO, USA). PCR reagents were from Applied Biosystems (Foster City, CA, USA). Fetal bovine serum was from Biochrom (Berlin, Germany). Other common reagents were from Sigma (St. Louis, MO, USA).

### 2.2. Mouse Models of Liver Injury

Mouse models of liver injury were induced by BDL. BDL was performed on male ICR mice (30.0 ± 1.0 g, six weeks age, *n* = 6). Sham-operated mice, used as controls, underwent a laparotomy with exposure, but no ligation of the common bile duct was performed. Mice were sacrificed at 7/14 days of BDL. For scRNA-seq, hepatocytes were isolated from one BDL mouse or one Sham mouse. All animal work was conformed to the Ethics Committee of Capital Medical University and in accordance with the approved guidelines (approval number AEEI-2014-131).

### 2.3. Mouse Primary Hepatocytes Preparation

Primary murine hepatocytes were isolated as previous research [9] and were used for immunofluorescence, qPCR and Western blot. For in vitro experiments, isolated mouse hepatocytes were cultured in William’s Medium E (Gibco, Life Technologies, Foster City, CA, USA) with 10% FBS on 24-well collagen-coated plate for four hours. Hepatocytes were incubated in the presence or absence of lipopolysaccharide (LPS, 100 ng/mL), and then the cells were used for qPCR.

### 2.4. Single-Cell RNA Sequencing

scRNA-seq was performed by Capitalbio Technology Corporation (Beijing, China). Cell suspensions were loaded on a Chromium Single Cell Controller (10× Genomics, San Francisco, CA) to generate single-cell gel beads in emulsion, following the manufacture’s introduction of Single Cell 3′ Library and Gel Bead Kit V2 (10× Genomics). Following Drop-seq droplet collection, cDNA amplification and sequencing library preparation were carried out exactly as described previously [22], and the libraries were sequenced on an Illumina HiSeq X Ten. For Drop-seq data from normal and cholestatic cells, the libraries from one batch of droplets were sequenced individually.

### 2.5. scRNA-Seq Data Analysis

Data analysis was mainly performed by Capitalbio Technology Corporation (Beijing, China). We used Cell Ranger 2.0.1 to analyze the sequencing data and generated the single cell information. Cell Ranger also provided pre-built mouse (mm10-1.2.0) reference packages for read alignment which finished by STAR-2.5.1b. For analysis of mix cells, the cells of different samples were merged together by Cell Ranger aggr pipeline and normalized by equalizing the read depth among libraries. Principal-component analysis and t-distributed Stochastic Neighbor Embedding (t-SNE) were performed using the prcomp and Rtsne package of the R software (Version 3.4.1). Pseudotime analysis was performed using Monocle 2 [23]. Gene hierarchical cluster was performed by Cluster 3.0.

### 2.6. Gene Ontology (GO) and Pathway Analysis

GO analysis and pathway analysis were performed using STRING database (https://string-db.org/). Benjamini & Hochberg adjusted *p*-value < 0.05 was recommended to present significantly differential the term.

### 2.7. Immunofluorescence

Primary hepatocytes were fixed in 4% paraformaldehyde and made into smear. Immunofluorescence was performed as previous described [9]. Albumin antibody (1:100, Santa Cruz Biotechnology, Santa Cruz, CA, USA), CD31 antibody (1:50, Santa Cruz Biotechnology, Santa Cruz, CA, USA), Laminin antibody (1:100, Abcam, Cambridge, UK), and Nestin antibody (1:50, Chemicon, Billerica, MA, USA) were used. FITC-conjugated donkey anti-goat antibody or Cy3-conjugated goat anti-rabbit antibody was used as secondary antibodies (1:100, Jackson Immuno-Research, West Grove, PA). At last, nuclei were stained with DAPI.

### 2.8. qPCR

qPCR was performed as described previously [9]. All primers were synthesized by Biotech (Beijing, China). Primers used for qPCR were as follows: 18S rRNA: sense, 5′-GTAACCCGTTGAACCCCATT-3′; antisense, 5′-CCATCCAATCGGTAGTAGCG-3′. *Ccl8*: sense, 5′-TACGCAGTGCTTCTTTGCCTG-3′; antisense, 5′-TTATCTGGCCCAGTCAGCTTCTC-3′. *Laminin*: sense, 5′-ATGTTTAGTGGGGGCGATG-3′; antisense, 5′-AGCGGTAGCGTTCAAAGGT-3′. *Hgf*: sense, 5′-AGCACCATCAAGGCAAGGT-3′; antisense, 5′-GACCAGGAACAATGACACCA-3′. *Cdh1*: sense, 5′-ATCCTCGCCCTGCTGATT-3′; antisense, 5′-ACCACCGTTCTCCTCCGTA-3′. *Cx43*: sense, 5′-TGTGCCCACACTCCTGTACTTG-3′; antisense, 5′-TTTCTTGTTCAGCTTCTCTTCCTTT-3′.

### 2.9. Western Blot

Western blot analysis was carried out with standard procedures and followed primary antibodies against Laminin (1:2000, Abcam, Cambridge, UK), HGF (1:2000, Abcam, Cambridge, UK), and Cx43 (1:1000, Sigma, St. Louis, MO, USA). IRDyeTM 800-conjugated goat anti-rabbit IgG (1:10,000, LI-COR Biosciences, Lincoln, NE, USA) was applied as secondary antibodies. Protein expression was visualized and quantified by the LI-COR Odyssey^®^ Imaging System and Odyssey^®^ software (LI-COR Biosciences, Lincoln, NE, USA), respectively. Results were normalized relative to β-Tubulin (1:1000; Cell Signaling, Beverly, MA, USA) expression to correct for variations in protein loading and transfer.

### 2.10. Statistical Analysis

The results are expressed as mean ± SEM from at least three independent experiments performed. Statistical significance was assessed by Student’s t-test or ANOVA for analysis of variance when appropriate. Correlation coefficients were calculated by a Pearson test. *p* < 0.05 was considered to be significant.

## 3. Results

### 3.1. Cholestasis-Injured Hepatocytes are Heterogeneous, Separating in Six Distinct Clusters

**Figure 1:**
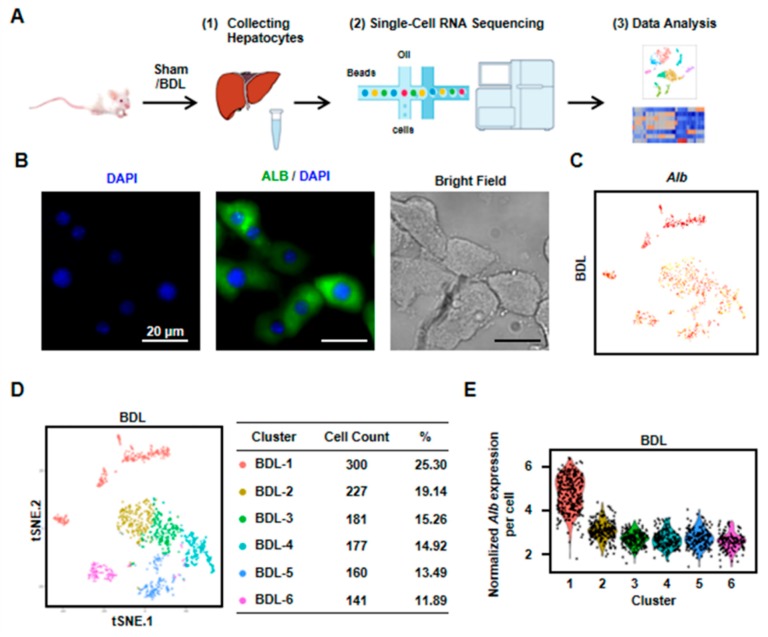
Six clusters were classified of cholestatic hepatocytes depending on scRNA-seq analysis. (**A**) Workflow depicts isolation of hepatocytes from liver for generating scRNA transcriptome profiles. (**B**) The staining of albumin (Alb) on isolated cholestatic hepatocyte smear. Scale bars, 20 μm. (**C**) The expressions of Alb in all cells were shown. (**D**) 2D visualization of single-cell clustering of hepatocytes profiles inferred from RNA-seq data. Six major classes of hepatocytes in injured liver were detected. The count of each cell population was indicated. Colored bar coded as indicated. (**E**) The expression of Alb in each cluster was shown.

To identify the heterogeneity and variation of hepatocytes in cholestasis-injured liver, BDL injury model was performed. After two weeks, we isolated hepatocytes from a mouse liver with BDL treatment and performed scRNA-seq (Figure 1A). We first employed immunofluorescence to detect the purity of isolated hepatocytes. The result showed that almost all cells expressed albumin (Alb, the marker of hepatocytes). At the same time, there are almost no NPCs in the isolated cells. These results indicated the isolated cells were hepatocytes with high purity (Figure 1B). Then, scRNA-seq was performed by 10× Genomics. The 10× Genomics sequenced the resultant single-cell transcriptomes to an average depth of more than 300,000 reads per cell (median genes per cell: 3303). We obtained single-cell transcriptomes from 1186 cells derived from mouse BDL liver (Figure 1C,D, Table S1). All the cells expressed *Alb*, indicating these obtained cells were all hepatocytes (Figure 1C). To clarify the differences in cell populations captured in our experiments, we selected the highly variable genes and performed hierarchical clustering on the significant principal components and visualized the cell clusters with t-SNE. Six cell clusters as cholestatic hepatocytes were obtained (named as BDL-1 to 6) (Figure 1D). We also found that *Alb* level in cholestatic hepatocyte clusters were different. *Alb* expression in BDL-1 cells was high while other five clusters were *Alb*-low hepatocytes (Figure 1C,E).

### 3.2. Hepatocytes undergo Gene Expression Profile Change after Liver Injury

**Figure 2:**
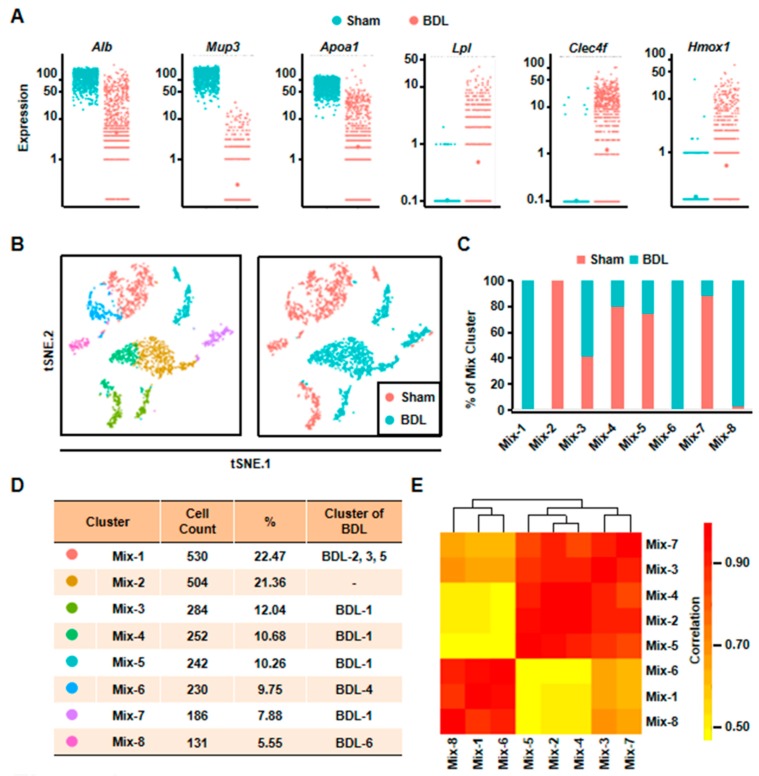
Eight clusters were classified of all the cells depending on scRNA-seq analysis. (**A**) The expressions of representative genes in normal and cholestatic hepatocytes were shown. (**B**) 2D visualization of single-cell clustering of hepatocytes profiles inferred from RNA-seq data in the mixed cells. Eight major classes of hepatocytes in normal and injured liver were detected. Colored bar coded as indicated in Figure 2D. (**C**) The components of each mix clusters. (**D**) The count of each cell population was indicated. Colored bar coded as indicated. (**E**) Correlation analysis of eight clusters.

To understand the changes of hepatocytes after liver injury, we isolated normal hepatocytes from one Sham mouse and compared gene expression profile between normal and cholestatic hepatocytes. We obtained 1173 single cell transcriptome data from normal hepatocytes (Table S1 and Figure S1). First, some known representative gene expressions were studied (Figure 2A). *Alb* was down-regulated after liver injury. Major urinary protein 3 (*Mup3*), which regulates glucose metabolism and is considered as another marker of hepatocytes, was also decreased. Furthermore, apolipoprotein a1 (*Apoa1*), which plays a role in lipid transfer, was reduced. However, the expression of lipoprotein lipase (*Lpl*), participating in lipid regulation, was increased after liver injury. At the same time, the inflammation related gene, C-type lectin domain family 4, member f (*Clec4f*), and heme oxygenase 1 (*Hmox1*) were up-regulated (Figure 2A). These results proved that hepatocyte gene expression profile was changed, suggesting functional change of hepatocytes in cholestasis.

Second, we analyzed all the 2359 hepatocytes from BDL and Sham mouse together. The result of t-SNE analysis clearly identified eight clusters (Figure 2B). Among these clusters, Mix-1 was the largest group which included BDL-2, BDL-3, and BDL-5. Mix-2 cells were all normal hepatocytes. The cells of Mix-6 were from BDL-4 hepatocytes, and most cells of the Mix-8 were from BDL-6 hepatocytes. All of the remaining hepatocyte clusters (Mix-3/4/5/7) were composed by BDL-1 cells and normal hepatocytes (Figure 2C,D). Correlation analysis also indicated that these eight clusters could be correlatively separated into two big groups. One was composed by Mix-1, Mix-6, and Mix-8. Cells belonged to the three clusters were from BDL-2 to 6. Another one was comprised by Mix-2, Mix-3, Mix-4, Mix-5, and Mix-7. The five clusters were composed of almost all of the normal hepatocytes and BDL-1 cells (Figure 2E). These results showed that BDL-1 hepatocytes were similar to normal hepatocytes, which indicated that BDL-1 was a cluster of “normal” hepatocytes injured less.

**Figure 3:**
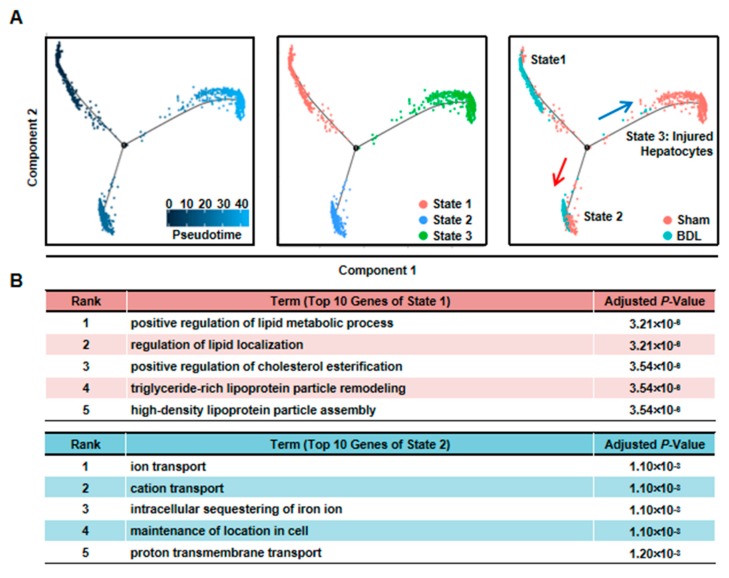
Pseudotime analysis indicated hepatocyte function transformation during liver injury. (**A**) Pseudotime analysis of hepatocytes was performed in the mixed cells. (**B**) GO analysis of top 10 genes of State 1 and State 2.

Third, we performed pseudotime analysis on the mixed cells. Pseudotime analysis is usually used to contribute cell developmental trajectory based on transcriptional similarities [23]. Here, we employed it to further study the changes of hepatocytes after injury. The result showed that these cells were separated into three different states. State 1/2 cells were from BDL and Sham sample, while State 3 cells were mainly from BDL sample (Figure 3A). Meanwhile, BDL hepatocytes belonged to State1/2 were from BDL-1 cluster. This result further indicated that BDL-1 hepatocytes were similar with normal hepatocytes. At the same time, the cells belonged to cluster 2-6 of BDL sample composed state 3 cells, which we named as “injured hepatocytes”. Since State 1 and State 2 cells were all normal hepatocytes, we asked what the difference was between these two states of hepatocytes. The top-10 most expressed genes of these two states were used to perform Gene Ontology (GO) analysis (Table S2). The results showed that hepatocytes belonged to State 1 mainly performed lipid metabolic functions, while State 2 hepatocytes were involved in transport (Figure 3B). These data proved that normal hepatocytes were heterogeneity and performed different functions.

### 3.3. Hepatocytes Responsible for Liver Repair are Identified

To further define the functions of cholestatic hepatocytes, the top-30 most expressed genes (absolute value of Log2 fold change ≥ 1, Benjamini & Hochberg adjusted *p*-value < 0.05) of each BDL clusters were selected for GO analysis. Among these clusters, we defined a cluster of cells (BDL-6) which were involved in tissue repair.

**Figure 4:**
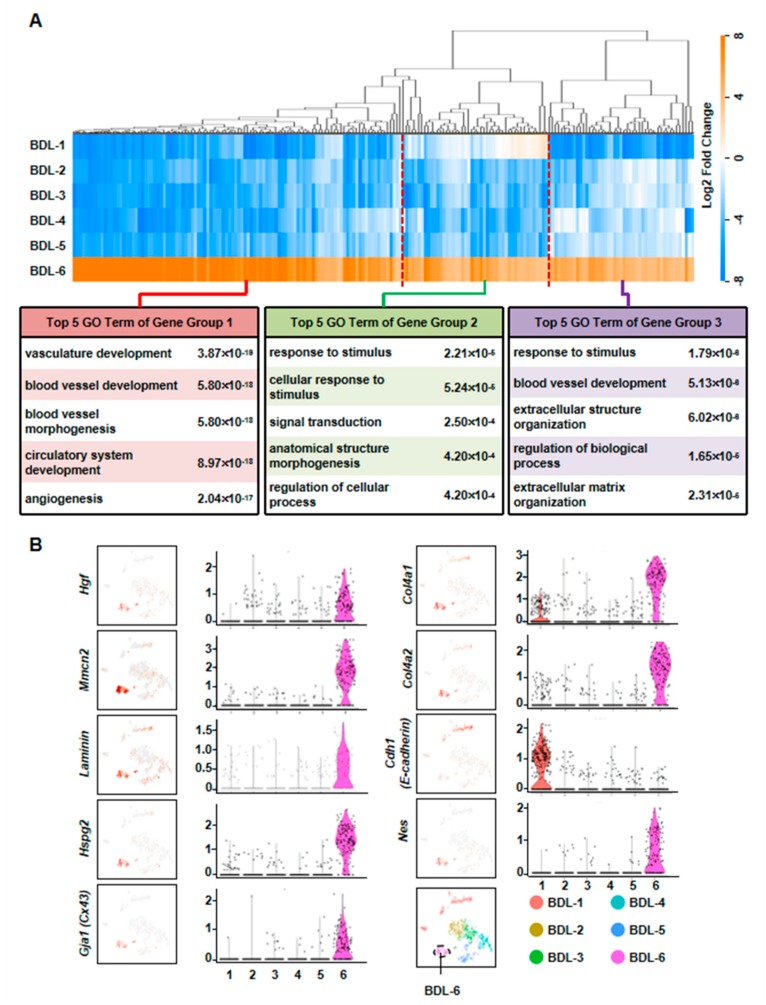
Elucidation of hepatocyte clusters participating in tissue repair. (**A**) Hierarchical cluster and GO analysis of Top 200 high expressed genes of BDL-6 in BDL sample. (**B**) Enrichment pattern of genes in the BDL-6.

To further clarify the functions of these hepatocytes during liver repair, we first chose more highly expressed genes of BDL-6 (Top 200) to perform hierarchical cluster. The result showed that all these 200 genes were divided into three gene groups (Figure 4A). Then, we analyzed the functions of the three gene groups by GO analysis. There were 113 genes belonged to Gene Group 1 and these genes were angiogenesis-related gene, suggesting the important role of hepatocytes in angiogenesis. The 40 genes belonged to Gene Group 2 were mainly involved in cellular response to stimulus and signal transduction. The top 5 GO terms of Gene Group 3 (47 genes) included extracellular structure organization and ECM organization, indicating these hepatocytes were involved in ECM reorganization after liver injury (Figure 4A).

The expressions of representative genes were also analyzed. In BDL-6 hepatocytes, multimerin 2 (*Mmrn2*) and *Hgf* were highly expressed (Figure 4B, Table S3). The two genes are important mediators of angiogenesis [24,25]. Furthermore, *Hgf* is also a factor improving liver regeneration and inducing EMT of liver tumor cells [26,27]. On the other hand, the expressions of ECM genes were also detected in this cluster, such as laminin, collagen type IV alpha 1 (*Col4a1*), *Col4a2*, and heparan sulfate proteoglycan 2 (*Hspg2*) (Figure 4B).

**Figure 5:**
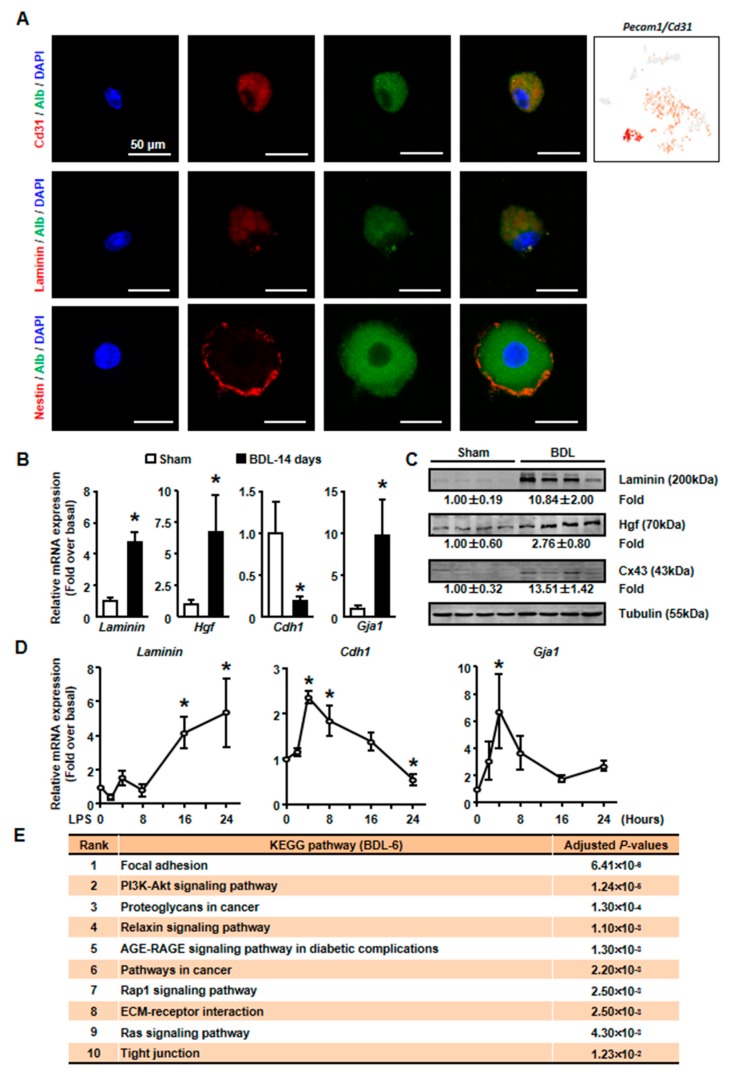
The expressions of tissue repair-related genes were changed in isolated cholestatic hepatocyte. (**A**) The detection of Alb and CD31, Laminin, Nestin on cholestatic hepatocyte smear. Scale bars, 50 μm. (**B**) The mRNA expressions of representative tissue repair-related genes were examined in isolated normal and cholestatic livers. (**C**) Western blot was employed to study the protein level of tissue repair-related genes. (**D**) Isolated normal hepatocytes were cultured with 100 ng/mL LPS and the mRNA expression of Laminin, E-cadherin and Cx43 were detected by qPCR. (**E**) Pathway analysis of top 200 high expressed genes of BDL-6. Data are presented as the means ± SEM. *p < 0.05 vs. control (*n* = 7 for each group in Figure 5B, *n* = 3 for each group in Figure 5D).

Then, isolated primary hepatocytes were used to confirm these results. Since we detected the expression of endothelial cells (ECs) marker, *Pecam1* (also known as Cd31), in BDL-6 cells (Figure 5A), we first asked whether these cells formed hepatocytes-EC pair during scRNA-seq [28]. We employed immunofluorescence assay to detect Cd31 expression on isolated cholestatic hepatocyte smear. Hepatocytes with Cd31^+^ signal were found on smear, while hepatocyte-EC pair was not found (Figure 5A). The expressions of representative genes were also detected in isolated hepatocytes. The results of qPCR and Western blot showed that laminin and *Hgf* expressions were increased in cholestatic hepatocytes (Figure 5B,C). Next, we treated primary hepatocytes with LPS to induce hepatocyte injury and found that laminin and *Hgf* expressions were also up-regulated in LPS-treated hepatocytes (Figure 5D).

We also employed pathway analysis to study the mechanism under the formation and function of tissue repair-related hepatocytes. The results showed that various signaling pathways might involve in these processes, including PI3K-AKT, Relaxin, AGE-RAGE, Rap1, and Ras signaling pathways (Figure 5E).

Taken together, hepatocytes involving in repair of liver injury (especially angiogenesis) were defined. Our results indicated the important role of hepatocytes during cholestatic liver injury.

### 3.4. The Liver Repair–Related Hepatocytes undergo EMT during Liver Injury

As mentioned earlier, the expressions of ECM genes and EMT-related gene (*Hgf*) were specifically detected in BDL-6 hepatocytes. Since ECM production is one of the features of hepatocyte EMT, we asked whether BDL-6 hepatocytes occurred EMT during liver injury. First, the expressions of EMT marker genes were studied in scRNA-seq. Nestin (*Nes*) and gap junction protein alpha-1 (*Gja1*, also named as Cx43) were highly expressed, while cadherin 1 (*Cdh1*, also known as E-cadherin) was lowly expressed (Figure 4B). Then, we examined whether EMT-occurring hepatocytes could be detected in cholestatic hepatocytes smear. Laminin and Nestin, which were high expressed in BDL-6, were chosen as marker genes and were detected by immunofluorescence. The results showed that Nestin^+^Alb^+^ or Laminin^+^Alb^+^ cell was existed (Figure 5A). Finally, the results of qPCR and Western blot showed that expressions of Laminin, Hgf, and Cx43 were increased in cholestatic and LPS-treated hepatocytes, while E-cadherin level was decreased (Figure 5B–D). In brief, these data proved that hepatocytes underwent EMT during liver injury.

### 3.5. Hepatocytes are Important Players in Liver Inflammation During Cholestatic Injury

**Figure 6:**
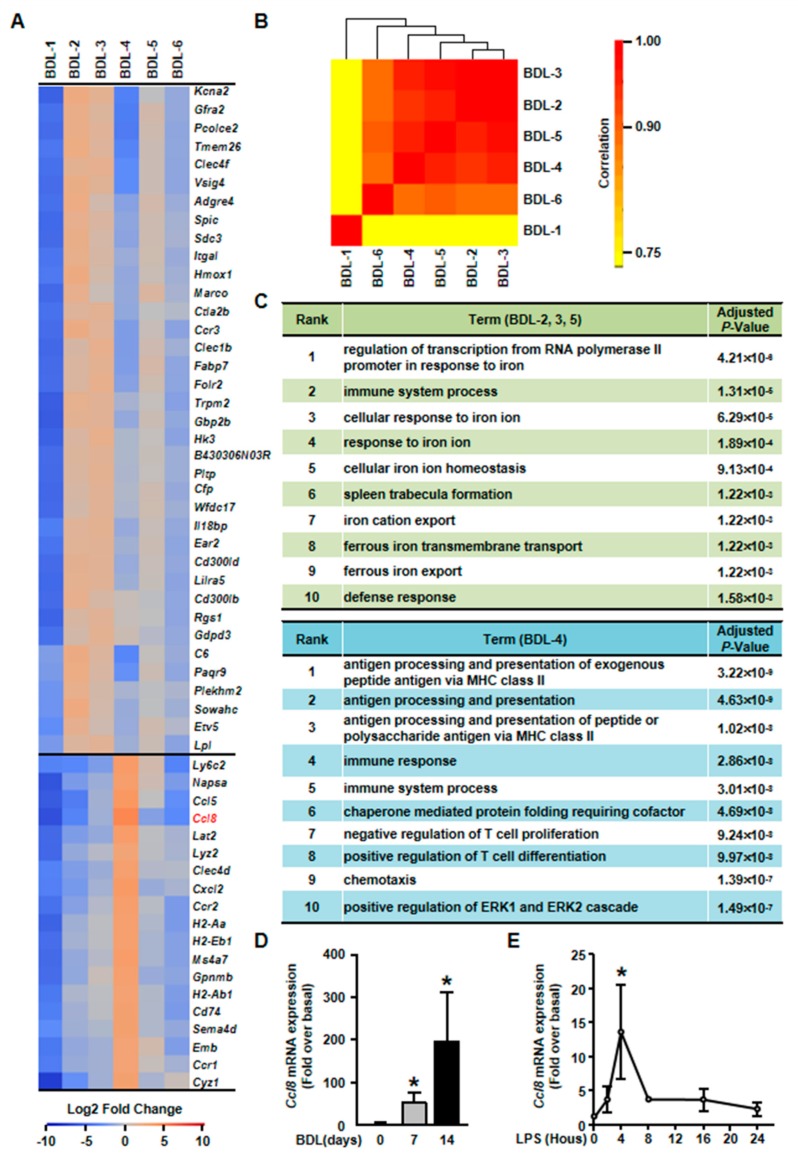
Elucidation of hepatocyte clusters participating in inflammation. (**A**) Heatmap of highly expressed genes of BDL-2, BDL-3, BDL-4 and BDL-5 in BDL sample. (**B**). Correlation analysis of cholestatic hepatocyte clusters. (**C**) GO enrichment analysis of BDL-2/3/5 and BDL-4. (**D**) Hepatocytes were isolated from Sham or BDL mouse livers and Ccl8 expression was detected. (**E**) Ccl8 expression in LPS-treated primary hepatocytes. Data are presented as the means ± SEM. *p < 0.05 vs. control (*n* = 7 for each group in Figure 6D, *n* = 3 for each group in Figure 6E).

It has been reported that hepatocytes are important players of liver inflammation during liver injury. We then studied the inflammatory functions of hepatocytes in our scRNA-seq data. Among cholestatic hepatocytes clusters, BDL-2/3/4/5 were involved in immune process (Figure 6C). However, these four inflammation-related clusters showed different gene expression profiles and functions.

First, gene heatmap showed that BDL-2/3/5 shared similar gene expression profiles (Figure 6A). We also performed correlation analysis for all cholestatic hepatocyte clusters to confirm this conclusion. The result showed that BDL-3 were highly correlated with BDL-2 and BDL-5 (Figure 6B). Herein, we merged these three clusters for further analysis. The top signature genes of BDL-2/3/5 includes *Clec4f*, V-set and immunoglobulin domain containing 4 (*Vsig4*), integrin alpha L (*Itgal*), *Hmox1*, and IL-18 binding protein (*Il18bp*) (Figure 6A, Table S4). All these genes were related to immune system process. For example, *Vsig4* is reported to regulate inflammatory factor expressions negatively [29]. *Hmox1*, who is reported as an anti-fibrogenetic protein in liver fibrosis, also functions as a modulator of inflammation and enhances autophagy [30,31,32]. Through the analysis of gene functions, we considered BDL-2/3/5 cells as inflammation-regulating hepatocytes, who were involved in liver inflammation positively or negatively.

**Figure 7:**
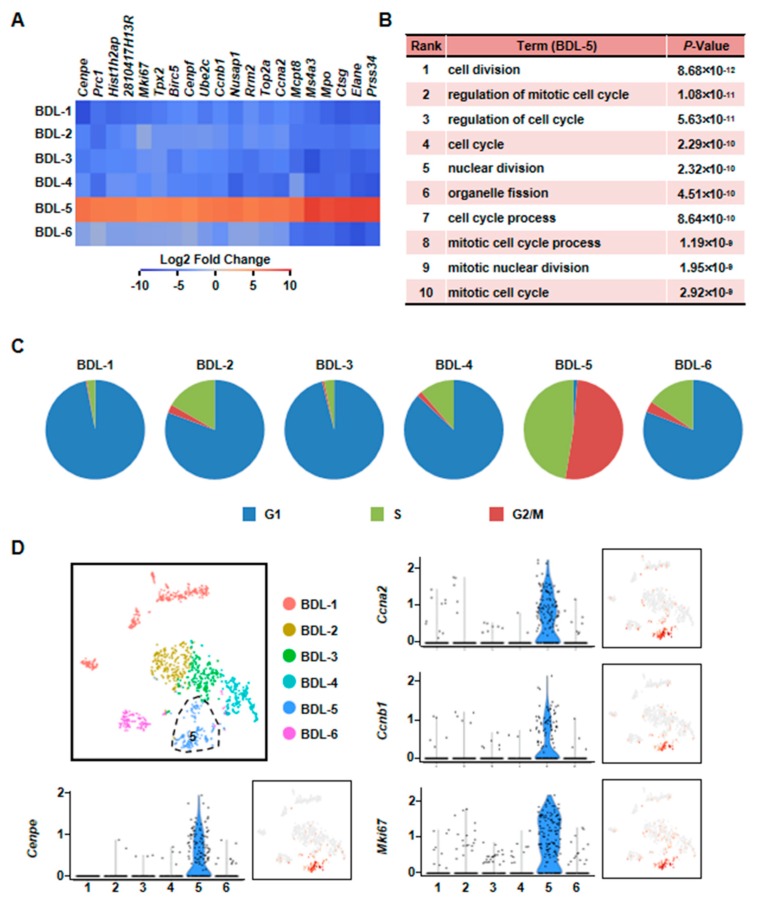
Characterization of proliferative hepatocyte cluster. (**A**) Heatmap showed top 20 highly expressed genes of BDL-5. (**B**) GO analysis of BDL-5 highly expressed genes. (**C**) Cell cycle analysis of six clusters of BDL sample. (**D**) Enrichment pattern of genes in BDL-5.

Second, we found that BDL-5 was a specific cluster in the three clusters. BDL-5 cells specifically expressed genes that regulate cell division and cell cycle, such as centromere protein E (*Cenpe*), nucleolar and spindle associated protein 1 (*Nusap1*), antigen identified by monoclonal antibody Ki 67 (*Mki67*), cyclin A2 (*Ccna2*) and cyclin B1 (*Ccnb1*) (Figure 7, Table S4) [33,34,35,36]. BDL-5 also distinguished from other clusters by GO terms associated with cell cycle and cell division (Figure 7B). The results of cell cycle analysis confirmed this conclusion, since cells of BDL-5 were almost proliferating cells (Figure 7C). Taken together, the cells belonging to BDL-5 were proliferative hepatocytes.

Third, BDL-4 was different from BDL-2/3/5 as cells in these cluster expressed chemokines and their receptors, such as *Ccl5*, *Ccl8*, and *Cxcl*2 (Figure 6A, Table S4). Owing to chemokine expression, GO analysis showed that the terms of chemotaxis regulation were enriching in this cluster (Figure 6C). Overall, the analysis indicated that BDL-4 hepatocytes significantly regulated leukocyte migration and affected immune function. We also performed qPCR to detect the expression of representative gene (*Ccl8* was chosen). The results of qPCR showed that *Ccl8* expression was up-regulated in cholestatic or LPS-treated hepatocytes (Figure 6D,E). These results illustrated that hepatocytes are important players in liver inflammation during cholestatic liver injury.

### 3.6. Characterization of a Less Damage Hepatocyte Cluster in Cholestatic Injured Liver

Final, we analyzed the characters of BDL-1 hepatocytes. BDL-1 was the biggest cluster of cholestatic hepatocytes and contained 300 cells (25.3%) (Figure 1D). Despite BDL-1 hepatocytes were isolated from injured liver, they still highly expressed some genes of normal hepatocytes, including choline kinase alpha (*Chka*), *Alb*, *Mup3*, and *Apoa1* (Figure S2A,C). The top discriminative genes also included glucose metabolism related genes, such as glucose-6-phosphatase (*G6pc*), solute carrier family 2 member 2 (*Slc2a2*) and solute carrier family 22 member 30 (*Slc22a30*) (Figure S2A,C, Table S5). According to GO enrichment analysis, BDL-1 distinctness was driven by terms associated with liver development and bile acid metabolic process (Figure S2B). All these results indicated that hepatocytes of BDL-1 were injured less and were similar with normal hepatocytes.

## 4. Discussion

In the current study, we studied the role of hepatocytes during cholestatic liver injury. Through scRNA-seq, we identified six clusters of cholestatic hepatocytes in BDL-treated mouse liver. BDL-1 hepatocytes expressed some genes which highly expressed in normal hepatocytes, indicating that BDL-1 hepatocytes were less injured. BDL-2, BDL-3 and BDL-5 cells participated in immune system regulation. Furthermore, the genes regulating cell cycle and division were increased in BDL-5 hepatocytes. BDL-4 cells also played a role in immune, but its function focused on leukocyte chemotaxis. Moreover, BDL-6 hepatocytes highly expressed the marker genes of EMT and genes mediating tissue repair, especially angiogenesis. Taken together, these analyses proved that hepatocytes were important players in liver injury.

EMT is a pathological process which is well studied in cancer research, but not in liver injury. During liver fibrogenesis, hepatic stellate cells are well-known source of collagen, but some researches also prove that hepatocytes contribute to collagen production via undergoing EMT. It has been reported that EMT is occurred in TGF-β1 (a well-known fibrotic factor)-stimulated hepatocytes [37,38]. In vitro, hepatocytes with TGF-β1 treatment show mesenchymal morphology, lose epithelial marker gene expression (like E-cadherin), and express mesenchymal genes (for example, vimentin, fibronectin, ZO-1) [38,39]. In vivo, a research based on lineage tracing mouse proves that hepatocytes contribute to the population of FSP1-positive fibroblasts in liver fibrosis [37]. After undergoing EMT, hepatocytes become a kind of ECM-producing cell and are involved in liver fibrogenesis. However, there are also reports against this conclusion. In a study based on another lineage tracing mouse, the researchers do not find any hepatocyte undergo EMT [14]. Therefore, it is still a controversy whether EMT occurs during liver fibrosis. In our current study, our data showed that EMT was occurred in some hepatocytes (BDL-6) during liver cholestatic injury. We have three pieces of evidence to support our conclusion. (1) scRNA-seq data showed EMT marker gene expressions in BDL-6 hepatocytes. (2) We found EMT marker gene-expressed hepatocytes on isolated cholestatic hepatocyte smear. (3) The changes of EMT marker gene expressions were confirmed by qPCR and western blot using isolated cholestatic hepatocytes and LPS-treated hepatocytes. Our research proved the existence of EMT in liver injury and added the first scRNA-seq evidence for this controversial issue.

During liver injury, the formation of new blood vessels, sinusoidal remodeling, and changes in ECM composition/organization were observed. In our scRNA-seq data, we found BDL-6 hepatocytes were also involved in these processes. Angiogenesis, the sprouting of new vessels from preexisting ones, is an essential pathophysiological process required for embryogenesis, growth, regeneration, and wound healing [40]. It has been reported that liver injury and pathological angiogenesis are interdependent processes that occur in parallel. Hepatic stellate cells play a key role in angiogenesis [41,42,43,44]. Furthermore, many kinds of cells have been discovered participating in angiogenesis, including macrophages, dendritic cells and so on [45,46]. However, the function of hepatocytes on angiogenesis is still not clear. The scRNA-seq results defined BDL-6 hepatocytes highly expressed angiogenesis-related genes, which illustrated that hepatocytes might meditate angiogenesis. We noticed the highly expressed genes and functions of BDL-6 cells were similar with ECs, and cells of this cluster were Cd31^+^. Therefore, we asked whether cells belonged to this cluster were cell pair formed by hepatocytes and ECs, as a recent research reported [28]. We performed immunofluorescence and did not find a hepatocyte-EC cell pair. Instead, Cd31^+^Alb^+^ cells were found. Since NPCs (include ECs) had been removed when hepatocytes were isolated, our data suggested the transdifferentiation between hepatocytes and ECs, but the details and direction (hepatocytes to ECs, or ECs to hepatocytes) of the transition should be studied in the future. It should be noted that the percentage of Cd31^+^Alb^+^ cells was low on the isolated hepatocyte smear (three cells among 150 detected hepatocytes).

There are many immunocytes mediating liver injury and inflammation, such as neutrophils, macrophages, and nature killer cells [47]. Recently, more and more studies focus on the immunologic function of hepatocytes [5]. It has been reported that the injured hepatocytes could secrete pro-inflammatory cytokines, such as IL-33, which promotes liver injury and fibrogenesis directly [48]. Macrophage migration inhibitory factor is another inflammatory cytokine secreted by hepatocytes and plays a critical role in liver damage [7,9]. Our previous study has also shown that MCP-1 expression is increased in injured hepatocytes in vitro [9]. Therefore, hepatocytes are one of the important components in liver inflammation and are considered as effective therapeutic target of liver diseases. Depending on our analysis, inflammatory hepatocytes participated in inflammation via two different manners during cholestatic liver injury. Hepatocytes belonging to BDL-2, BDL-3, and BDL-5 highly expressed inflammation-related genes. BDL-4 hepatocytes, different from the three cluster cells, expressed chemotaxis-related genes and mediated immunocyte chemotaxis. In brief, our results proved that the inflammatory process mediated hepatocytes were complex since different hepatocytes performed quite different immunologic functions. Our data provided more information on hepatocyte-involved hepatic inflammation.

scRNA-seq has been used to study the heterogeneity of normal hepatocytes [18,19,20]. At present, liver zonation has been identified by scRNA-seq, and the liver is divided into nine layers with different gene expression profiles [20]. Recent studies based on a normal human liver further support this opinion [19,21]. In these studies, hepatocytes are divided into three groups—pericentral hepatocytes, periportal hepatocytes, and middle-layer hepatocytes. Since the localization of hepatocytes is critical for their response to injury, further analyses are needed to study the relationship between hepatocyte zonation and hepatocyte heterogeneity during cholestasis.

In summary, these comprehensive analyses provide first scRNA transcriptome profiles of cholestatic hepatocytes. The analyses show the heterogeneity of cholestatic hepatocyte, which may give rise to further study on hepatocyte function during liver injury. Therefore, our data provide much more information for future treatment of hepatic injury aiming at hepatocytes and open new perspectives for treatment of hepatic injury.

## Supplementary Materials

The following are available online at https://www.mdpi.com/2073-4409/8/9/1069/s1, Figure S1: Quality characterization of drop-seq scRNA-seq data. Figure S2: Characterization of a hepatocyte cluster less injured isolated from cholestasis injury liver. Table S1: metrics summary. Table S2: Top10 gene among state. Table S3: High expressed genes of BDL-6. Table S4: High expressed genes of BDL-2-5. Table S5: High expressed genes of BDL-1.

## Abbreviations

BDL	bile duct ligation
scRNA-seq	single-cell RNA sequencing
t-SNE	t-distributed Stochastic Neighbor Embedding
GO	Gene ontology
Alb	albumin
Clec4f	C-type lectin domain family 4 member f
Vsig4	V-set and immunoglobulin domain containing 4
Itgal	integrin alpha L
Hmox1	heme oxygenase 1
Il18bp	IL-18 binding protein
Nusap1	nucleolar and spindle associated protein 1
Mki67	monoclonal antibody Ki 67
Ccna2	cyclin A2
Ccnb1	cyclin B1
ECM	extracellular matrix
Mmrn2	multimerin 2
Col4a	collagen type IV alpha
Hspg2	heparan sulfate proteoglycan 2
EMT	epithelial-mesenchymal transition
Nes	Nestin
Gja1	gap junction protein alpha-1
Cdh1	cadherin 1
ECs	endothelial cells
Chka	choline kinase alpha
Mup3	major urinary protein 3
Apoa1	apolipoprotein A1
G6pc	glucose-6-phosphatase
Slc2a2	solute carrier family 2 member 2
Slc22a30	solute carrier family 22 member 30
NPCs	non-parenchymal cells
Lpl	lipoprotein lipase
