# Supplementary material for: Single-Cell Transcriptomes Reveal Characteristic Features of Mouse Hepatocytes with Liver Cholestatic Injury
Source: Cells. 2019 Sep 11;8(9):1069. doi: 10.3390/cells8091069 (PMC6770527; doi:10.3390/cells8091069)
Supplement: Supplementary file 1 [file cells-08-01069-s001.zip › supplemental figures and tables/Supplemental Table 1.pdf]

Single-cell transcriptomes reveal characteristic features of Mouse Hepatocytes with Cholestasis Injury

Na Chang, Lei Tian, Xiaofang Ji, Xuan Zhou, Lei Hou, Xinhao Zhao, Yuanru Yang, Lin Yang, Liying Li

Supplemental Table 1: metrics summary (Related to Supplementary Figure 1)

Sample	Estimated Number of Cells	Mean Reads per Cell	Median Genes per Cell	Number of Reads	Valid Barcodes	Reads Mapped Confidently to Transcriptome	Reads Mapped Confidently to Exonic Regions	Reads Mapped Confidently to Intronic Regions	Reads Mapped Confidently to Intergenic Regions	Reads Mapped Antisense to Gene	Sequencing Saturation	Q30 Bases in Barcode	Q30 Bases in RNA Read	Q30 Bases in UMI	Fraction Reads in Cells	Total Genes Detected	Median UMI Counts per Cell
Sham	1,173	361,220	968	423,712,047	97.80%	64.00%	66.40%	2.00%	1.30%	1.60%	51.40%	97.10%	89.60%	97.00%	3.70%	13,117	3,680
BDL	1,186	340,465	3,303	403,792,392	97.40%	71.00%	73.70%	7.10%	2.80%	2.70%	85.00%	96.90%	82.30%	96.80%	44.40%	17,538	12,542
