# Supplementary material for: Single-Cell Transcriptomes Reveal Characteristic Features of Mouse Hepatocytes with Liver Cholestatic Injury
Source: Cells. 2019 Sep 11;8(9):1069. doi: 10.3390/cells8091069 (PMC6770527; doi:10.3390/cells8091069)
Supplement: Supplementary file 1 [file cells-08-01069-s001.zip › supplemental figures and tables/Supplemental Table 2.pdf]

Single-cell transcriptomes reveal characteristic features of Mouse Hepatocytes with Cholestasis Injury

Na Chang, Lei Tian, Xiaofang Ji, Xuan Zhou, Lei Hou, Xinhao Zhao, Yuanru Yang, Lin Yang, Liying Li

Supplemental Table 2: Top10 gene among state (Related to Figure 3)

State	gene	symbol	log2Mean
1	ENSMUSG00000074115	Saa1	8.00
1	ENSMUSG00000057465	Saa2	7.93
1	ENSMUSG00000029368	Alb	6.94
1	ENSMUSG00000005681	Apoa2	6.77
1	ENSMUSG00000066154	Mup3	6.76
1	ENSMUSG00000031722	Hp	6.56
1	ENSMUSG00000030895	Hpx	6.38
1	ENSMUSG00000078672	Mup20	6.22
1	ENSMUSG00000032083	Apoa1	6.17
1	ENSMUSG00000002985	Apoe	6.08
2	ENSMUSG00000049775	Tmsb4x	7.21
2	ENSMUSG00000050708	Ftl1	7.03
2	ENSMUSG00000002985	Apoe	6.41
2	ENSMUSG00000024661	Fth1	6.18
2	ENSMUSG00000092341	Malat1	6.09
2	ENSMUSG00000064351	mt-Co1	5.85
2	ENSMUSG00000064357	mt-Atp6	5.73
2	ENSMUSG00000064358	mt-Co3	5.66
2	ENSMUSG00000029580	Actb	5.16
2	ENSMUSG00000034892	Rps29	5.16
