# Supplementary material for: Single-Cell Transcriptomes Reveal Characteristic Features of Mouse Hepatocytes with Liver Cholestatic Injury
Source: Cells. 2019 Sep 11;8(9):1069. doi: 10.3390/cells8091069 (PMC6770527; doi:10.3390/cells8091069)
Supplement: Supplementary file 1 [file cells-08-01069-s001.zip › supplemental figures and tables/Supplemental Table 3.pdf]

Single-cell transcriptomes reveal characteristic features of Mouse Hepatocytes with Cholestasis Injury

Na Chang, Lei Tian, Xiaofang Ji, Xuan Zhou, Lei Hou, Xinhao Zhao, Yuanru Yang, Lin Yang, Liying Li

Supplemental Table 3: High expressed genes of BDL-6 (Related to Figure 4)

Top 200 hige expressed genes of BDL-6

Gene Group	EnsemblID	GeneName	Cluster 1 Average	Cluster 1 Log2 Fold Change	Cluster 1 P-Value	Cluster 2 Average	Cluster 2 Log2 Fold Change	Cluster 2 P-Value	Cluster 3 Average	Cluster 3 Log2 Fold Change	Cluster 3 P-Value	Cluster 4 Average	Cluster 4 Log2 Fold Change	Cluster 4 P-Value	Cluster 5 Average	Cluster 5 Log2 Fold Change	Cluster 5 P-Value	Cluster 6 Average	Cluster 6 Log2 Fold Change	Cluster 6 P-Value
Group 1	ENSMUSG00000039167	Adgrl4	0.057549	-4.7628	1.45E-12	0.079169	-4.10188	1.30E-03	0.026784	-5.72084	1.10E-10	0.05536	-4.69239	1.33E-07	0.056994	-4.69961	2.13E-09	7.730521	7.16226	4.63E-64
	ENSMUSG00000051855	Mest	0.010156	-4.68855	8.71E-08	0.013195	-3.97297	1.96E-02	0.007653	-4.88755	4.84E-06	0.01384	-4.1447	2.27E-04	0.012213	-4.35995	2.32E-05	1.626105	7.086081	3.88E-40
	ENSMUSG00000058396	Gpr182	0.023697	-5.15241	1.58E-12	0.046182	-4.01805	2.44E-03	0.03061	-4.76134	2.14E-08	0.032293	-4.60856	6.48E-07	0.044781	-4.2334	1.28E-07	4.469615	6.992164	1.03E-57
	ENSMUSG00000032125	Robo4	0.010156	-4.74008	2.05E-10	0.013195	-4.02437	3.26E-03	0.007653	-4.93895	7.78E-08	0.009227	-4.61488	2.17E-06	0.028497	-3.39695	1.37E-05	1.673932	6.847683	3.74E-52
	ENSMUSG00000056492	Adgrf5	0.027082	-5.11107	1.29E-13	0.052779	-3.97671	1.33E-03	0.03061	-4.89128	2.66E-09	0.032293	-4.73838	8.64E-08	0.073278	-3.69198	4.44E-07	4.865271	6.828116	2.31E-61
	ENSMUSG00000062960	Kdr	0.06432	-5.34015	3.31E-15	0.059377	-5.21056	7.57E-05	0.080352	-4.98191	6.15E-10	0.087653	-4.7963	2.55E-08	0.146555	-4.11589	2.11E-08	12.77405	7.149553	7.82E-68
	ENSMUSG00000041378	Cldn5	0.037238	-5.02564	2.97E-13	0.032987	-4.8972	2.35E-04	0.045915	-4.68939	6.85E-09	0.050747	-4.48179	3.24E-07	0.069207	-4.10613	6.49E-08	6.143546	6.975366	6.24E-62
	ENSMUSG00000054690	Emcn	0.010156	-4.92321	1.56E-10	0.006597	-4.7952	1.34E-03	0.011479	-4.70342	2.02E-07	0.009227	-4.79758	2.07E-06	0.020355	-4.00326	1.87E-06	1.917413	7.223701	3.50E-53
	ENSMUSG00000044562	Rasip1	0.023697	-4.48786	5.38E-11	0.013195	-4.77926	6.03E-04	0.026784	-4.26807	1.34E-07	0.018453	-4.62852	4.97E-07	0.036639	-3.83301	6.05E-07	2.813075	6.775649	2.09E-56
	ENSMUSG00000003411	Rab3b	0.013541	-4.34015	1.91E-07	0.006597	-4.53913	9.17E-03	0.015305	-4.12037	2.35E-05	0.01384	-4.12204	1.83E-04	0.032568	-3.14821	8.12E-04	1.569583	6.450218	3.41E-37
	ENSMUSG00000022579	Gpihbp1	0.060934	-4.81625	6.83E-13	0.052779	-4.7671	2.81E-04	0.084178	-4.31794	3.16E-08	0.046133	-5.06666	2.08E-08	0.077349	-4.41293	8.21E-09	8.42618	6.978384	9.47E-63
	ENSMUSG00000037010	Apln	0.010156	-4.45299	2.08E-04	0.006597	-4.32742	1.51E-01	0.011479	-4.2332	3.70E-03	0.009227	-4.32858	1.18E-02	0.012213	-4.12438	7.10E-03	1.378276	6.955132	3.43E-23
	ENSMUSG00000074491	Clec4g	0.074475	-4.75183	5.69E-12	0.072572	-4.56291	7.90E-04	0.107136	-4.19388	1.56E-07	0.101493	-4.20798	2.03E-06	0.101775	-4.24448	7.94E-08	9.678368	6.695391	3.83E-56
	ENSMUSG00000001946	Esam	0.020311	-5.04737	5.08E-13	0.039585	-3.91301	1.75E-03	0.038263	-4.16882	8.02E-08	0.023067	-4.72758	1.48E-07	0.056994	-3.60581	9.75E-07	3.60004	6.6456	7.20E-58
	ENSMUSG00000052921	Arhgef15	0.013541	-4.34015	6.29E-10	0.013195	-3.95037	2.84E-03	0.022958	-3.62729	3.23E-06	0.009227	-4.54088	1.78E-06	0.020355	-3.7447	2.25E-06	1.573931	6.524587	4.30E-51
	ENSMUSG00000034684	Sema3f	0.020311	-4.35318	3.78E-10	0.02639	-3.70964	4.65E-03	0.038263	-3.47047	6.34E-06	0.023067	-4.03442	6.75E-06	0.036639	-3.50188	5.58E-06	2.195677	6.1964	2.01E-48
	ENSMUSG00000001240	Ramp2	0.023697	-5.35487	2.49E-14	0.052779	-4.0494	1.14E-03	0.065047	-3.95331	1.55E-07	0.0692	-3.80154	3.85E-06	0.04071	-4.56329	3.07E-09	5.091361	6.679465	7.76E-60
	ENSMUSG00000009654	Oit3	0.016926	-4.30278	2.24E-09	0.019792	-3.7599	6.57E-03	0.026784	-3.66142	6.57E-06	0.02768	-3.53327	8.29E-05	0.020355	-3.97418	1.81E-06	1.826107	6.305478	2.38E-46
	ENSMUSG00000027358	Bmp2	0.027082	-4.25634	8.73E-09	0.046182	-3.29416	2.12E-02	0.034437	-3.88231	6.57E-06	0.046133	-3.41849	2.64E-04	0.028497	-4.09991	3.09E-06	2.643508	6.20067	2.10E-42
	ENSMUSG00000044258	Ctla2a	0.088016	-4.81154	4.11E-13	0.131949	-4.0428	1.30E-03	0.160704	-3.9122	2.04E-07	0.239893	-3.28134	5.15E-05	0.227975	-3.38956	3.28E-06	11.561	6.097848	3.59E-52
	ENSMUSG00000025964	Adam23	0.033852	-4.3598	4.88E-10	0.039585	-3.88434	4.20E-03	0.053568	-3.68569	3.63E-06	0.087653	-2.93797	5.85E-04	0.056994	-3.57687	6.50E-06	3.430473	5.968398	1.25E-44
	ENSMUSG00000025608	Podxl	0.027082	-4.25634	3.25E-09	0.02639	-3.97894	4.94E-03	0.022958	-4.40362	5.33E-07	0.004613	-5.89806	1.30E-07	0.028497	-4.09991	1.36E-06	2.708726	6.873204	1.07E-50
	ENSMUSG00000041445	Mmrn2	0.033852	-5.42095	3.83E-13	0.052779	-4.57773	1.11E-03	0.049742	-4.85078	1.92E-08	0.018453	-6.01951	6.26E-09	0.048852	-4.8497	8.54E-09	7.482692	7.507596	3.98E-62
	ENSMUSG00000046916	Myct1	0.027082	-4.34354	4.50E-10	0.039585	-3.57598	6.90E-03	0.042089	-3.70236	2.80E-06	0.009227	-5.39729	1.27E-07	0.028497	-4.18698	2.94E-07	2.847858	6.570958	1.37E-51
	ENSMUSG00000033191	Tie1	0.020311	-4.10947	8.36E-10	0.02639	-3.46692	5.46E-03	0.03061	-3.5207	3.19E-06	0.009227	-4.80075	4.45E-07	0.032568	-3.41188	5.32E-06	1.86089	6.229542	1.44E-50
	ENSMUSG00000000093	Tbx2	0.016926	-4.02663	2.66E-08	0.019792	-3.48512	1.14E-02	0.011479	-4.39969	1.05E-06	0.004613	-5.08347	1.91E-06	0.024426	-3.47168	2.75E-05	1.526104	6.554193	2.77E-46
	ENSMUSG00000004655	Aqp1	0.233582	-4.29164	3.49E-12	0.250702	-4.00987	9.11E-04	0.19514	-4.50577	2.66E-09	0.1384	-4.93383	5.78E-09	0.341962	-3.67869	2.00E-07	21.36111	6.512614	1.08E-61
	ENSMUSG00000038007	Acer2	0.033852	-3.97803	2.06E-08	0.032987	-3.72925	8.59E-03	0.015305	-4.90907	1.20E-07	0.018453	-4.585	4.16E-06	0.032568	-3.94339	3.46E-06	2.717421	6.632708	5.83E-48
	ENSMUSG00000026921	Egfl7	0.216656	-3.69677	2.56E-10	0.178131	-3.7943	1.11E-03	0.126267	-4.42604	2.66E-09	0.092267	-4.80301	6.48E-09	0.175052	-3.94071	1.65E-08	13.13928	6.349866	3.32E-61
	ENSMUSG00000028763	Hspg2	0.108328	-3.02267	7.55E-08	0.059377	-3.64355	1.68E-03	0.107136	-2.99506	7.74E-06	0.046133	-4.09515	4.13E-07	0.101775	-3.04808	6.50E-06	4.034827	5.487978	4.70E-48
	ENSMUSG00000046470	Sox18	0.00677	-5.00272	1.17E-09	0.019792	-3.4493	1.66E-02	0.011479	-4.36387	2.17E-06	0.004613	-5.04784	4.34E-06	0.016284	-3.92909	9.23E-06	1.508712	6.978278	7.14E-47
	ENSMUSG00000042807	Hecw2	0.00677	-5.29295	5.99E-12	0.039585	-2.923	1.92E-02	0.015305	-4.32962	2.30E-07	0.009227	-4.7491	8.82E-07	0.024426	-3.72875	2.05E-06	1.826107	6.668048	7.60E-53
	ENSMUSG00000034911	Ushbp1	0.003385	-5.0187	3.33E-09	0.013195	-3.29341	2.00E-02	0.003826	-4.79892	1.31E-06	0.004613	-4.47485	2.38E-05	0.012213	-3.67813	2.77E-05	1.017403	7.049281	7.36E-46
	ENSMUSG00000027955	Fam198b	0.00677	-4.53724	1.92E-08	0.02639	-2.65478	6.74E-02	0.011479	-3.89687	1.57E-05	0.01384	-3.5728	2.53E-04	0.024426	-2.96388	4.71E-04	1.056533	6.025499	2.19E-38
	ENSMUSG00000020154	Ptprb	0.07109	-3.64041	3.09E-09	0.131949	-2.57457	3.20E-02	0.068873	-3.63635	8.04E-07	0.0692	-3.56442	1.17E-05	0.093633	-3.18345	8.22E-06	4.117437	5.616723	2.76E-46
	ENSMUSG00000036185	Sapcd1	0.013541	-3.97758	1.35E-03	0.02639	-2.84322	5.38E-01	0.003826	-5.09438	1.72E-03	0.009227	-4.18048	2.35E-02	0.016284	-3.64898	2.71E-02	1.226101	6.487347	1.72E-19

ENSMUSG00000032135	Mcam	0.016926	-3.51488	5.31E-06	0.02639	-2.64918	9.49E-02	0.007653	-4.3119	1.54E-05	0.004613	-4.57836	6.42E-05	0.016284	-3.45493	2.39E-04	1.060881	6.191864	7.94E-36
ENSMUSG00000000317	Bcl6b	0.020311	-3.88494	4.28E-08	0.039585	-2.75058	3.94E-02	0.011479	-4.48368	6.64E-07	0.01384	-4.15962	1.81E-05	0.048852	-2.64065	7.87E-04	1.569583	5.888339	4.58E-41
ENSMUSG00000029648	Flt1	0.010156	-4.69975	3.57E-07	0.02639	-3.23974	8.07E-02	0.011479	-4.47997	3.78E-05	0.032293	-3.14095	5.68E-03	0.036639	-3.02674	3.21E-03	1.582626	6.099554	1.44E-30
ENSMUSG00000049807	Arhgap23	0.06432	-2.88644	5.00E-07	0.092364	-2.18012	6.61E-02	0.061221	-2.90894	2.12E-05	0.08304	-2.4192	1.30E-03	0.142484	-1.6674	1.77E-02	2.034805	4.529633	2.05E-33
ENSMUSG00000040283	Btnl9	0.013541	-3.78354	1.51E-06	0.013195	-3.39733	3.29E-02	0.007653	-4.3119	1.54E-05	0.023067	-2.97103	2.78E-03	0.008142	-4.20308	2.53E-05	1.065229	6.285204	2.07E-36
ENSMUSG00000029762	Akr1b8	0.040623	-3.55422	4.52E-08	0.032987	-3.55295	6.90E-03	0.034437	-3.72052	2.33E-06	0.050747	-3.12837	2.11E-04	0.020355	-4.3587	1.51E-07	2.343504	5.99324	6.48E-46
ENSMUSG00000053062	Jam2	0.013541	-4.59169	3.64E-07	0.006597	-4.78885	1.20E-02	0.022958	-3.88006	1.40E-04	0.009227	-4.79121	1.20E-04	0.008142	-5.00645	9.94E-06	1.904369	7.120766	8.00E-38
ENSMUSG00000036256	Igfbp7	0.771836	-2.71879	3.16E-07	0.659743	-2.79787	9.94E-03	1.02927	-2.25084	3.16E-04	0.85808	-2.47797	4.60E-04	0.643215	-2.93434	5.04E-06	21.36111	4.725788	9.86E-15
ENSMUSG00000022469	Rapgef3	0.020311	-3.81605	4.66E-08	0	-5.51237	6.39E-04	0.015305	-4.08951	1.83E-06	0.009227	-4.51019	5.71E-06	0.020355	-3.71375	7.63E-06	1.547843	6.652544	3.37E-48
ENSMUSG00000041134	Cyyr1	0.023697	-3.95471	5.13E-09	0.006597	-4.83892	8.94E-04	0.03061	-3.56188	5.41E-06	0.032293	-3.41086	7.43E-05	0.024426	-3.82186	1.82E-06	1.913065	6.220433	7.40E-48
ENSMUSG00000073599	Ecscr	0.023697	-3.79333	8.64E-08	0.006597	-4.67973	2.17E-03	0.015305	-4.26203	1.61E-06	0.02768	-3.4456	1.92E-04	0.028497	-3.46472	3.10E-05	1.71741	6.271606	8.97E-44
ENSMUSG00000026399	Cd55	0.06432	-3.22293	1.55E-07	0.019792	-4.44324	9.36E-04	0.026784	-4.3497	8.02E-08	0.050747	-3.43251	3.95E-05	0.073278	-2.97039	4.76E-05	2.856553	5.82199	2.38E-46
ENSMUSG00000028776	Tinagl1	0.230197	-2.69695	2.45E-06	0.065974	-4.26094	6.24E-04	0.080352	-4.1663	3.20E-08	0.05536	-4.60877	7.64E-08	0.073278	-4.2715	8.06E-09	6.991382	5.97227	3.12E-52
ENSMUSG00000040605	Bace2	0.040623	-2.63302	2.80E-05	0.013195	-3.66129	7.58E-03	0.011479	-4.15618	1.54E-06	0.009227	-4.2518	1.26E-05	0.024426	-3.22598	5.64E-05	1.24784	5.812175	2.88E-41
ENSMUSG00000020357	Flt4	0.07109	-2.97356	1.09E-06	0.032987	-3.74902	3.62E-03	0.034437	-3.91788	6.64E-07	0.046133	-3.45411	4.51E-05	0.036639	-3.80906	9.60E-07	2.656551	5.818717	1.57E-45
ENSMUSG00000032766	Gng11	0.331754	-3.19883	1.06E-08	0.171533	-3.96923	6.12E-04	0.206619	-3.84563	3.77E-08	0.22144	-3.69073	1.36E-06	0.284969	-3.36169	5.56E-07	13.88711	5.76997	9.98E-54
ENSMUSG00000020176	Grb10	0.067705	-2.1873	2.04E-04	0.02639	-3.18658	1.26E-02	0.034437	-3.08169	3.87E-05	0.032293	-3.08737	2.44E-04	0.024426	-3.49916	7.77E-06	1.434799	5.158788	5.51E-37
ENSMUSG00000027210	Meis2	0.084631	-2.0859	3.11E-04	0.013195	-4.14539	1.96E-03	0.019131	-4.04998	5.38E-07	0.032293	-3.30418	8.20E-05	0.044781	-2.92098	7.14E-05	1.678279	5.266454	2.68E-39
ENSMUSG00000007039	Ddah2	0.128639	-2.36133	1.55E-05	0.072572	-2.97857	9.46E-03	0.049742	-3.66703	3.34E-07	0.078427	-2.9729	8.96E-05	0.134342	-2.24028	8.30E-04	2.926119	4.919115	1.74E-39
ENSMUSG00000049804	Armcx4	0	-7.28471	4.15E-14	0.072572	-2.53786	6.12E-02	0.022958	-4.24263	6.85E-07	0.01384	-4.73341	1.78E-06	0.044781	-3.34362	2.73E-05	2.391331	6.448598	7.43E-48
ENSMUSG00000040732	Erg	0.003385	-5.22912	8.82E-11	0.019792	-3.08442	1.70E-02	0.015305	-3.67187	6.25E-06	0.009227	-4.09515	1.39E-05	0.056994	-1.92504	9.93E-03	1.113056	5.704458	8.87E-42
ENSMUSG00000050493	Fam167b	0.016926	-5.82435	1.18E-15	0.118754	-3.01217	1.11E-02	0.10331	-3.35695	2.39E-06	0.02768	-5.05697	1.74E-08	0.048852	-4.37229	6.45E-09	5.239189	6.470179	4.17E-58
ENSMUSG00000011263	Exoc3l2	0.003385	-5.55105	2.95E-11	0.02639	-3.08234	1.94E-02	0.038263	-2.83425	1.84E-04	0.01384	-3.99893	2.38E-05	0.024426	-3.39434	2.64E-05	1.417407	6.05198	1.38E-43
ENSMUSG00000024065	Ehd3	0.033852	-4.84008	1.11E-11	0.184728	-2.28492	9.36E-02	0.118615	-3.05373	4.49E-05	0.08304	-3.49789	5.23E-05	0.118058	-3.04052	6.65E-05	4.647878	5.53572	9.07E-41
ENSMUSG00000045377	Tmem88	0.00677	-4.9285	2.80E-10	0.032987	-2.7814	3.20E-02	0.015305	-3.96327	2.39E-06	0.004613	-4.97384	1.72E-06	0.032568	-2.98933	1.28E-04	1.400016	6.285762	6.48E-46
ENSMUSG00000024168	Tmem204	0.010156	-4.58768	1.88E-10	0.052779	-2.26316	7.07E-02	0.034437	-3.02165	3.49E-05	0.009227	-4.46288	2.03E-06	0.056994	-2.30729	1.34E-03	1.413059	5.481964	1.33E-41
ENSMUSG00000013076	Amotl1	0.016926	-4.43374	7.08E-11	0.032987	-3.29938	6.81E-03	0.042089	-3.19596	9.70E-06	0.01384	-4.4808	7.36E-07	0.048852	-2.96864	3.33E-05	1.965239	5.956603	4.45E-49
ENSMUSG00000045930	Clec14a	0.010156	-4.16593	8.96E-08	0.019792	-3.03157	3.70E-02	0.026784	-2.92445	3.75E-04	0.009227	-4.04249	7.99E-05	0.024426	-3.01373	4.10E-04	1.082621	5.849042	9.85E-37
ENSMUSG00000025666	Tmem47	0.016926	-3.67475	1.11E-07	0.032987	-2.54039	5.19E-02	0.038263	-2.55505	6.52E-04	0.009227	-4.14595	1.74E-05	0.032568	-2.74597	4.47E-04	1.130447	5.416399	2.41E-37
ENSMUSG00000004891	Nes	0.003385	-5.59968	4.90E-11	0.039585	-2.63791	5.25E-02	0.053568	-2.42025	1.49E-03	0.004613	-5.05584	2.03E-06	0.036639	-2.91692	2.65E-04	1.439146	5.717729	3.80E-39
ENSMUSG00000022468	Endou	0	-6.25971	1.46E-09	0.006597	-4.1203	1.59E-02	0.011479	-4.02472	4.32E-05	0.018453	-3.37361	1.31E-03	0.004071	-4.92606	5.29E-06	1.200013	7.134503	3.49E-40
ENSMUSG00000062515	Fabp4	0.081246	-5.39556	2.37E-14	0.197923	-3.94866	2.44E-03	0.187488	-4.16657	1.33E-07	0.40136	-3.01281	3.01E-04	0.199478	-4.05775	1.55E-07	16.13931	6.294168	3.59E-52
ENSMUSG00000040624	Plekhg1	0.003385	-5.68104	1.77E-12	0.013195	-3.95795	2.44E-03	0.038263	-2.96752	3.97E-05	0.04152	-2.78481	5.15E-04	0.020355	-3.75233	1.68E-06	1.547843	6.015115	2.00E-47
ENSMUSG00000068735	Trp53i11	0.013541	-4.13106	2.70E-09	0.046182	-2.30533	6.37E-02	0.049742	-2.38559	7.36E-04	0.046133	-2.42299	2.31E-03	0.032568	-2.9367	7.89E-05	1.252188	5.09135	6.92E-37
ENSMUSG00000059588	Calcrl	0.013541	-4.61711	1.22E-09	0.079169	-2.07882	1.52E-01	0.084178	-2.13786	5.52E-03	0.073813	-2.26943	9.58E-03	0.056994	-2.6717	8.48E-04	1.708715	4.856217	2.53E-30
ENSMUSG00000020689	Itgb3	0.013541	-4.13106	1.68E-08	0.052779	-2.13095	1.23E-01	0.076526	-1.76851	2.17E-02	0.078427	-1.68069	6.06E-02	0.044781	-2.5082	1.53E-03	1.173926	4.494101	4.56E-27
ENSMUSG00000020674	Pxdn	0.00677	-4.5483	2.01E-09	0.013195	-3.41394	1.07E-02	0.03061	-2.71013	2.59E-04	0.01384	-3.5839	7.31E-05	0.024426	-2.97511	1.33E-04	1.052186	5.80807	1.03E-41
ENSMUSG00000048960	Prex2	0.023697	-3.31901	8.41E-07	0.02639	-2.87717	3.19E-02	0.049742	-2.26253	2.69E-03	0.032293	-2.77516	1.47E-03	0.036639	-2.65876	7.89E-04	1.147839	5.034986	9.11E-33
ENSMUSG00000021759	Plpp1	0.074475	-4.71519	1.33E-13	0.197923	-3.14507	6.94E-03	0.183662	-3.38753	1.26E-06	0.15224	-3.60038	4.45E-06	0.24833	-2.93078	1.94E-05	9.052274	5.759806	6.45E-51
ENSMUSG00000024538	Ppic	0.020311	-4.05071	1.38E-09	0.039585	-2.91635	1.66E-02	0.045915	-2.91771	4.49E-05	0.064587	-2.38042	2.45E-03	0.08142	-2.08973	3.67E-03	1.652192	5.033693	3.16E-37
ENSMUSG00000018593	Sparc	0.575492	-4.05526	2.49E-13	1.233719	-2.78185	7.97E-03	1.49225	-2.61208	2.12E-05	1.642346	-2.42397	4.25E-04	1.766806	-2.34329	1.71E-04	40.1874	4.930151	9.86E-15
ENSMUSG00000006386	Tek	0.013541	-4.10434	6.73E-09	0.052779	-2.10389	1.02E-01	0.011479	-4.21097	8.37E-07	0	-5.90029	2.09E-07	0.020355	-3.5082	1.11E-05	1.321754	6.203037	3.22E-46
ENSMUSG00000024754	Tmem2	0.196344	-2.23261	1.40E-05	0.270495	-1.61208	1.76E-01	0.206619	-2.11968	5.24E-04	0.073813	-3.54133	1.56E-06	0.211691	-2.06707	1.07E-03	3.786999	4.316458	2.84E-34
ENSMUSG00000070348	Ccnd1	0.622885	-2.1081	8.33E-06	0.92364	-1.38674	2.29E-01	0.635163	-2.04469	2.77E-04	0.507466	-2.33664	2.38E-04	1.127662	-1.14615	8.82E-02	9.926197	3.721959	6.85E-29
ENSMUSG00000002504	Slc9a3r2	0.121869	-2.3354	6.91E-06	0.237507	-1.20104	4.17E-01	0.19514	-1.59325	1.09E-02	0.101493	-2.50613	3.42E-04	0.236117	-1.28734	6.57E-02	2.300026	3.720112	1.13E-25

ENSMUSG00000022475	Hdac7	0.115098	-1.25133	2.16E-02	0.105559	-1.23996	4.18E-01	0.053568	-2.34385	4.05E-04	0.092267	-1.50797	5.03E-02	0.08142	-1.72322	1.26E-02	1.04349	3.541333	1.89E-21
ENSMUSG00000066571	4931406P16Rik	0.2065	-1.30046	8.50E-03	0.211118	-1.1475	4.10E-01	0.114788	-2.15708	2.63E-04	0.12456	-1.98704	2.81E-03	0.16691	-1.58349	1.14E-02	1.908717	3.539086	8.71E-25
ENSMUSG00000054263	Lifr	0.399459	-0.95991	9.85E-02	0.158338	-2.22551	5.78E-02	0.210446	-1.92913	3.48E-03	0.19376	-2.00624	7.32E-03	0.175052	-2.18679	1.30E-03	3.039164	3.649242	3.21E-22
ENSMUSG00000025790	Slco3a1	0.2065	-1.19549	2.26E-02	0.072572	-2.56043	1.81E-02	0.126267	-1.91672	2.22E-03	0.175307	-1.38172	6.74E-02	0.134342	-1.8079	5.78E-03	1.80002	3.572051	3.19E-23
ENSMUSG00000032966	Fkbp1a	2.247803	-0.92107	4.18E-02	1.319486	-1.6487	7.32E-02	1.446335	-1.59767	2.01E-03	2.195946	-0.91317	1.94E-01	1.774948	-1.26653	2.56E-02	13.95233	2.917575	9.86E-15
ENSMUSG00000020315	Sptbn1	0.958024	-0.71662	1.40E-01	0.508002	-1.60786	9.13E-02	0.497417	-1.73462	9.62E-04	0.516693	-1.63743	6.05E-03	0.769415	-1.04373	9.92E-02	5.360929	2.982459	1.77E-20
ENSMUSG00000039081	Zfp503	0.013541	-3.844	2.97E-07	0.006597	-4.04763	7.91E-03	0.007653	-4.37191	3.54E-06	0.023067	-3.03172	1.17E-03	0.052923	-1.98069	1.96E-02	1.069577	5.590617	5.24E-35
ENSMUSG00000063415	Cyp26b1	0.003385	-5.26976	7.28E-08	0	-5.14041	8.59E-03	0.007653	-4.46	3.74E-05	0.018453	-3.38888	2.56E-03	0.016284	-3.60413	4.82E-04	1.204361	6.876668	1.91E-35
ENSMUSG00000074785	Plxnc1	0.044008	-4.21432	6.57E-10	0.039585	-4.09034	2.44E-03	0.061221	-3.70997	2.39E-06	0.147627	-2.4049	3.99E-03	0.142484	-2.49006	1.06E-03	3.826129	5.439187	2.04E-39
ENSMUSG00000020044	Timp3	0.044008	-4.00673	6.49E-10	0.059377	-3.36465	7.04E-03	0.049742	-3.78695	7.39E-07	0.17992	-1.90295	1.83E-02	0.08142	-3.08109	2.55E-05	3.295689	5.34079	6.12E-41
ENSMUSG00000004044	Ptrf	0.081246	-2.60319	3.27E-06	0.105559	-2.04544	8.52E-02	0.099483	-2.26728	5.60E-04	0.17992	-1.34259	9.32E-02	0.150626	-1.63705	2.02E-02	1.947848	3.99339	7.59E-27
ENSMUSG00000071042	Rasgrp3	0.037238	-3.76043	2.50E-09	0.006597	-5.23461	1.94E-04	0.011479	-5.1445	3.27E-09	0.147627	-1.70635	3.20E-02	0.061065	-3.00725	2.61E-05	2.41307	5.48435	5.86E-44
ENSMUSG00000029581	Fscn1	0.016926	-4.2428	2.08E-09	0.032987	-3.10844	1.72E-02	0.011479	-4.61477	1.81E-07	0.147627	-1.14459	2.43E-01	0.052923	-2.66432	4.41E-04	1.608713	4.995322	3.50E-34
ENSMUSG00000072235	Tuba1a	0.148951	-2.53872	3.91E-06	0.145143	-2.40481	3.40E-02	0.118615	-2.82988	2.12E-05	0.438266	-0.82434	4.35E-01	0.256472	-1.67361	1.64E-02	3.321776	3.922005	5.46E-26
ENSMUSG00000017309	Cd300lg	0.010156	-4.25466	7.15E-09	0.013195	-3.54039	9.53E-03	0.007653	-4.45497	7.77E-07	0.0692	-1.64875	5.52E-02	0.069207	-1.68346	3.44E-02	1.078273	4.980763	4.70E-33
ENSMUSG00000054435	Gimap4	0.057549	-4.29076	6.54E-13	0.098961	-3.32852	2.43E-03	0.088005	-3.64934	8.42E-08	0.29064	-1.86547	9.00E-03	0.370459	-1.52496	2.50E-02	4.878315	4.761586	6.27E-40
ENSMUSG00000032035	Ets1	0.081246	-3.66909	1.71E-10	0.131949	-2.79112	1.07E-02	0.130094	-2.95167	6.34E-06	0.336773	-1.49882	4.71E-02	0.301253	-1.69344	1.19E-02	4.282656	4.467954	6.05E-35
ENSMUSG00000047867	Gimap6	0.010156	-5.29213	1.61E-13	0.032987	-3.56787	0.002655	0.049742	-3.24012	4.14E-06	0.2076	-1.11724	0.199541	0.109916	-2.09574	0.001997	2.14785	4.739385	1.85E-36
ENSMUSG00000030653	Pde2a	0.050779	-4.12359	7.81E-13	0.131949	-2.58997	0.012015	0.126267	-2.79118	6.57E-06	0.350613	-1.22584	0.103763	0.346033	-1.2683	0.065148	3.591344	4.188943	2.08E-33
ENSMUSG00000043505	Gimap5	0.010156	-4.43969	9.65E-09	0.02639	-2.97894	0.033634	0.034437	-2.87101	0.000331	0.133787	-0.86827	0.485864	0.113987	-1.13726	0.279124	1.100012	4.121921	4.12E-22
ENSMUSG00000022440	C1qtnf6	0.037238	-2.75328	4.96E-06	0.019792	-3.2416	0.009936	0.049742	-2.30152	0.001035	0.101493	-1.2173	0.163796	0.122129	-0.9617	0.306073	1.013055	3.891126	8.73E-24
ENSMUSG00000038587	Akap12	0.016926	-4.19083	1.76E-08	0.032987	-3.05647	0.030249	0.026784	-3.54894	2.43E-05	0.106107	-1.58205	0.096036	0.016284	-4.12879	3.4E-06	1.604366	5.382218	1.47E-34
ENSMUSG00000032060	Cryab	0.077861	-1.90719	0.014488	0.105559	-1.30008	0.741581	0.141572	-0.96305	0.528018	0.147627	-0.86474	0.635226	0.069207	-2.01917	0.057339	1.013055	3.235774	2.31E-10
ENSMUSG00000067925	Cxx1a	0.192959	-1.46344	0.002064	0.151741	-1.68201	0.100581	0.214272	-1.27117	0.034679	0.419813	-0.16942	1	0.219833	-1.2187	0.05845	1.617409	2.74617	1.24E-15
ENSMUSG00000024074	Crim1	0.260664	-1.14311	0.016898	0.448625	-0.20568	1	0.290797	-0.94419	0.16199	0.156853	-1.84877	0.00365	0.240188	-1.23285	0.052083	1.760889	2.708817	2.74E-15
ENSMUSG00000079018	Ly6c1	0.088016	-3.3006	2.84E-05	0.059377	-3.62369	0.038539	0.022958	-5.05712	1.84E-06	0.80272	0.172414	0.919512	0.044781	-4.16356	3.72E-05	3.443516	4.147318	3.86E-17
ENSMUSG00000019647	Sema6a	0.088016	-1.72433	0.002831	0.019792	-3.44121	0.007852	0	-6.3679	2.66E-09	0.023067	-3.43863	6.74E-05	0.016284	-3.92098	1.3E-06	1.39132	5.383716	1.05E-39
ENSMUSG00000045092	S1pr1	0.368992	-0.27268	0.775406	0.046182	-3.18948	0.003803	0.011479	-5.11368	6.15E-10	0.09688	-2.28638	0.001133	0.073278	-2.72053	2.64E-05	1.939152	3.833937	5.68E-27
ENSMUSG00000024501	Dpysl3	0.14218	-1.18533	0.040377	0.019792	-3.62369	0.004604	0.022958	-3.72016	1.73E-06	0.078427	-1.9934	0.012015	0.077349	-2.04922	0.004666	1.386972	4.205186	3.55E-27
ENSMUSG00000000058	Cav2	0.162492	-0.81156	0.171969	0.039585	-2.66195	0.02426	0.026784	-3.3799	4.75E-06	0.0692	-2.0236	0.008058	0.097704	-1.55783	0.031244	1.16523	3.75594	2.59E-23
ENSMUSG00000007655	Cav1	0.07109	-1.84986	0.03192	0.006597	-4.26926	0.040227	0.03061	-2.9816	0.005427	0.170693	-0.43735	1	0.065136	-1.91739	0.123134	1.013055	3.820737	1.77E-12
ENSMUSG00000020186	Csrp2	0.541639	-0.38547	0.722916	0.039585	-4.03311	0.005041	0.076526	-3.34105	3.46E-05	0.37368	-0.95333	0.452713	0.321607	-1.20753	0.24824	2.600029	3.13031	1.16E-12
ENSMUSG00000058254	Tspan7	0.081246	-3.10883	7.9E-07	0.059377	-3.32303	0.010666	0.011479	-5.57006	2.66E-09	0.01384	-5.24599	1.36E-07	0.048852	-3.74499	2.05E-06	3.378298	6.245876	1.04E-48
ENSMUSG00000021944	Gata4	0.084631	-1.90322	0.001454	0.039585	-2.73555	0.033616	0.015305	-4.14309	1.11E-06	0.01384	-4.1447	1.05E-05	0.012213	-4.35995	4.11E-07	1.504365	5.389164	4.23E-38
ENSMUSG00000031503	Col4a2	0.240352	-1.81713	0.000488	0.118754	-2.68334	0.01352	0.141572	-2.57024	5.04E-05	0.046133	-4.07384	2.4E-07	0.097704	-3.08448	2.88E-06	3.726128	4.756606	2.18E-39
ENSMUSG00000031502	Col4a1	0.517942	-1.64041	0.001135	0.296884	-2.3337	0.026274	0.352018	-2.19655	0.000278	0.087653	-4.14545	2.93E-08	0.215762	-2.90205	3.4E-06	6.878337	4.467309	3.39E-37
ENSMUSG00000022893	Adamts1	0.209885	-1.22585	0.079705	0.065974	-2.73967	0.072368	0.042089	-3.52632	4.03E-05	0.023067	-4.21658	3.8E-05	0.077349	-2.6612	0.001931	2.191329	4.567941	1.10E-24
ENSMUSG00000051236	Msrb3	0.13541	-0.88695	0.113723	0.059377	-1.93817	0.098889	0.026784	-3.18384	7.92E-06	0.023067	-3.28391	4.45E-05	0.04071	-2.60182	0.000159	1.091316	4.150044	5.81E-29
ENSMUSG00000026181	Ppm1f	0.223426	-0.59217	0.315631	0.131949	-1.29768	0.32672	0.072699	-2.28605	0.000218	0.046133	-2.85478	7.08E-05	0.113987	-1.61022	0.01303	1.300014	3.401583	5.07E-22
ENSMUSG00000024238	Zeb1	0.230197	-0.30873	0.683442	0.118754	-1.24503	0.355382	0.088005	-1.80289	0.003491	0.08304	-1.83554	0.008287	0.089562	-1.75943	0.006115	1.030446	3.008688	2.91E-17
ENSMUSG00000038145	Snrk	0.582262	-0.51852	0.342274	0.395846	-1.05294	0.417699	0.317581	-1.47358	0.006843	0.253733	-1.77377	0.003292	0.329749	-1.40237	0.015298	2.804379	2.857901	2.41E-18
ENSMUSG00000010047	Hyal2	0.399459	-0.35984	0.600032	0.303482	-0.73732	0.788299	0.19514	-1.49477	0.011615	0.106107	-2.34824	0.000369	0.170981	-1.68036	0.005566	1.769585	2.880688	9.94E-17
ENSMUSG00000017615	Tnfaip1	0.572106	-0.39089	0.520006	0.415638	-0.83892	0.633488	0.348192	-1.18622	0.040348	0.19376	-2.03511	0.000755	0.317537	-1.31764	0.024875	2.421766	2.672395	6.28E-16
ENSMUSG00000021756	Il6st	1.130672	-0.27883	0.743389	0.69273	-1.0074	0.546964	0.70021	-1.0604	0.113824	0.31832	-2.22599	0.000607	0.549582	-1.42861	0.023418	4.452223	2.652354	6.35E-14

	ENSMUSG00000028517	Plpp3	1.11036	-0.56603	0.316673	0.613561	-1.41441	0.205978	0.470633	-1.90814	0.000635	0.08304	-4.36334	6.35E-10	0.309395	-2.51987	1.03E-05	6.552247	3.581438	3.77E-27
	ENSMUSG00000030352	Tspan9	0.352065	-0.19937	0.85113	0.138546	-1.56404	0.142711	0.19514	-1.15403	0.066403	0.04152	-3.32417	2.17E-06	0.154697	-1.49706	0.014274	1.434799	2.906022	1.66E-17
	ENSMUSG00000031217	Efnb1	0.230197	-0.41758	0.548506	0.250702	-0.22697	1	0.091831	-1.83611	0.003206	0.08304	-1.93011	0.00606	0.085491	-1.92283	0.003085	1.052186	2.858111	4.34E-15
	ENSMUSG00000045954	Sdpr	0.203115	-1.90042	0.001317	0.032987	-4.20229	0.001565	0.026784	-4.69864	2.6E-08	0.0692	-3.36173	6.77E-05	0.036639	-4.26469	9.61E-08	3.534822	5.411661	2.31E-40
	ENSMUSG00000074457	S100a16	0.582262	-0.84388	0.118982	0.092364	-3.42163	0.001215	0.065047	-4.06988	2.66E-09	0.115333	-3.20649	5.19E-06	0.08142	-3.73537	8E-09	4.7044	4.463505	1.24E-37
	ENSMUSG00000033174	Mgll	0.324983	-0.80599	0.152978	0.052779	-3.28941	0.00293	0.061221	-3.27035	1.26E-06	0.0692	-3.03577	3.76E-05	0.061065	-3.25102	1.15E-06	2.513071	4.281704	1.17E-32
	ENSMUSG00000027800	Tm4sf1	0.189574	-1.54772	0.012586	0.019792	-4.36422	0.0022	0.053568	-3.34821	2.02E-05	0.02768	-4.14095	9.57E-06	0.024426	-4.3562	3.12E-07	2.573942	5.128313	2.14E-34
Group 2	ENSMUSG00000037166	Ppp1r14a	0.301287	-0.46907	0.530186	0.039585	-3.25616	0.007039	0.084178	-2.41178	0.000373	0.078427	-2.45484	0.001342	0.077349	-2.5127	0.000328	1.73915	3.723361	2.01E-22
	ENSMUSG00000029710	Ephb4	0.233582	-0.72878	0.235227	0.059377	-2.589	0.026274	0.019131	-4.2522	7.78E-08	0.009227	-4.93681	1.27E-07	0.012213	-4.73413	5.23E-09	1.795672	4.560443	2.05E-33
	ENSMUSG00000032548	Slco2a1	0.453623	-0.63035	0.291514	0.112156	-2.60452	0.013861	0.126267	-2.57559	3.6E-05	0.050747	-3.78961	5.91E-07	0.089562	-3.04858	2.21E-06	3.026121	4.01952	8.21E-30
	ENSMUSG00000030551	Nr2f2	0.473934	-0.57316	0.365944	0.138546	-2.32303	0.034281	0.19514	-1.9478	0.002004	0.04152	-4.07011	2.73E-07	0.085491	-3.12879	2.3E-06	2.956555	3.829388	4.21E-26
	ENSMUSG00000027803	Wwtr1	0.247123	-0.20327	0.882116	0.032987	-2.97297	0.010284	0.057394	-2.43465	0.000211	0.02768	-3.33735	2.86E-05	0.032568	-3.18251	6.62E-06	1.230448	3.737549	4.13E-24
	ENSMUSG00000027663	Zmat3	0.578877	0.061983	0.932432	0.158338	-1.89527	0.063265	0.172183	-1.88983	0.001081	0.101493	-2.60986	8.48E-05	0.130271	-2.28526	0.000122	2.134806	3.080633	2.62E-19
	ENSMUSG00000024140	Epas1	0.788762	0.252857	0.584912	0.184728	-1.98094	0.040489	0.229577	-1.77269	0.001568	0.156853	-2.29089	0.0003	0.134342	-2.54951	9.94E-06	2.508723	2.913989	5.35E-18
	ENSMUSG00000058173	Smco4	1.015573	0.182496	0.717827	0.184728	-2.409	0.01877	0.149225	-2.847	3.97E-06	0.106107	-3.27823	3.22E-06	0.362317	-1.50941	0.019258	3.521778	3.099357	2.15E-18
	ENSMUSG00000031523	Dlc1	0.988491	0.232281	0.624104	0.092364	-3.29819	0.001138	0.072699	-3.79175	5.98E-09	0.036907	-4.63281	1.18E-09	0.08142	-3.61134	7.1E-09	3.778303	3.639955	8.88E-27
	ENSMUSG00000041261	Car8	0.517942	0.813801	0.064951	0.039585	-3.04687	0.011277	0.034437	-3.43771	3E-06	0.01384	-4.45384	8.32E-07	0.024426	-3.85263	4.38E-07	1.313058	3.11013	1.05E-15
	ENSMUSG00000030516	Tjp1	0.436697	0.38337	0.419133	0.032987	-3.33471	0.00293	0.015305	-4.51523	6.85E-09	0.018453	-4.19116	3.9E-07	0.028497	-3.7196	1.33E-07	1.526104	3.573302	2.76E-23
	ENSMUSG00000035184	Fam124a	0.470549	0.810276	0.052246	0.052779	-2.54039	0.019993	0.026784	-3.62825	4.49E-07	0.023067	-3.72591	3.41E-06	0.048852	-2.80212	2.67E-05	1.152187	2.971707	5.34E-16
	ENSMUSG00000037712	Fermt2	0.744754	0.619285	0.125666	0.112156	-2.32694	0.01767	0.107136	-2.53185	1.71E-05	0.073813	-3.00186	9.62E-06	0.069207	-3.13269	3.12E-07	1.991326	2.961038	5.30E-18
	ENSMUSG00000034738	Nostrin	0.504401	0.517955	0.242442	0.059377	-2.69612	0.010529	0.038263	-3.47047	2.99E-07	0.0692	-2.59216	0.000198	0.08142	-2.40129	0.000129	1.460886	3.066869	4.66E-18
	ENSMUSG00000015094	Npdc1	0.463779	0.247134	0.623892	0.052779	-2.93255	0.005041	0.061221	-2.90894	4.31E-06	0.08304	-2.4192	0.000406	0.052923	-3.088	1.53E-06	1.643496	3.316286	2.07E-21
	ENSMUSG00000023067	Cdkn1a	4.163851	0.386878	0.411315	0.738912	-2.32272	0.014989	0.742299	-2.42438	1.74E-05	0.807333	-2.25422	0.000347	0.66357	-2.57513	6.51E-06	12.47405	2.956849	9.86E-15
	ENSMUSG00000041801	Phlda3	0.826	0.553711	0.206157	0.072572	-3.12198	0.003309	0.099483	-2.84113	7.31E-06	0.087653	-2.96198	3.18E-05	0.109916	-2.67813	2.25E-05	2.343504	3.061953	1.72E-17
	ENSMUSG00000047907	Tshz2	0.47732	0.582303	0.183492	0.059377	-2.56836	0.015673	0.095657	-2.05613	0.000829	0.073813	-2.37241	0.000677	0.097704	-2.00696	0.001665	1.213057	2.717085	9.03E-14
	ENSMUSG00000028445	Enho	0.734598	0.926879	0.015252	0.079169	-2.5711	0.011068	0.053568	-3.27466	3.34E-07	0.115333	-2.13167	0.001258	0.020355	-4.5082	1.3E-10	1.613061	2.783816	1.98E-15
	ENSMUSG00000097312	Gm26870	0.934328	1.313464	0.1099	0.02639	-4.06982	0.567794	0.015305	-4.98439	0.014363	0.046133	-3.51019	0.186163	0.052923	-3.37115	0.15487	1.669584	2.664617	2.09E-03
	ENSMUSG00000041012	Cmtm8	1.123901	1.186782	0.00089	0.065974	-3.2676	0.001062	0.026784	-4.64659	1.1E-10	0.092267	-2.90851	1.05E-05	0.089562	-2.98914	5.12E-07	2.113067	2.655757	1.13E-14
Group 3	ENSMUSG00000027074	Slc43a3	0.027082	-4.21068	2.54E-11	0.046182	-3.24857	0.00468	0.080352	-2.67092	4.86E-05	0.073813	-2.73062	0.000243	0.350104	-0.41576	0.864879	2.134806	4.166263	1.24E-29
	ENSMUSG00000001029	Icam2	0.027082	-4.8338	6.91E-15	0.125351	-2.53077	0.014833	0.202793	-1.95964	0.001143	0.309093	-1.2807	0.085963	0.512944	-0.4968	0.745919	3.008729	3.681369	8.54E-26
	ENSMUSG00000016494	Cd34	0.010156	-5.00673	4.99E-10	0.032987	-3.28138	0.022382	0.03061	-3.60192	2.05E-05	0.239893	-0.57961	0.778509	0.056994	-2.73779	0.000923	1.73915	4.615108	1.59E-26
	ENSMUSG00000026656	Fcgr2b	0.094787	-4.53475	5.67E-15	0.178131	-3.4516	0.001108	0.306103	-2.80289	6.33E-06	2.232852	0.362333	0.634418	0.606576	-1.76401	0.005669	7.687042	3.544568	1.96E-23
	ENSMUSG00000000184	Ccnd2	0.081246	-4.74369	4.17E-16	0.389248	-2.32596	0.024422	0.474459	-2.14309	0.000316	1.102586	-0.80708	0.392771	0.586221	-1.80807	0.003709	8.404441	4.053047	1.84E-31
	ENSMUSG00000027435	Cd93	0.040623	-5.20917	1.78E-17	0.138546	-3.30857	0.001335	0.260187	-2.53558	2.4E-05	1.097973	-0.27075	0.982351	0.341962	-2.11255	0.000533	6.01311	4.049397	1.17E-31
	ENSMUSG00000001506	Col1a1	0.030467	-5.36756	0.039858	0.20452	-2.53439	1	0.260187	-2.30486	0.79675	0.9688	-0.22291	0.931729	0.439666	-1.49691	1	4.882663	3.739023	5.64E-04
	ENSMUSG00000029661	Col1a2	0.060934	-4.97862	0.030933	0.369456	-2.23457	1	0.378802	-2.30967	0.72436	1.416293	-0.21624	0.946812	0.708351	-1.34239	1	6.952251	3.631194	4.19E-04
	ENSMUSG00000025969	Nrp2	0.074475	-3.59309	3.01E-11	0.164936	-2.27767	0.02426	0.378802	-1.14159	0.082846	0.60896	-0.35558	0.879736	0.386743	-1.09767	0.125071	2.973946	3.215755	1.92E-20
	ENSMUSG00000030787	Lyve1	0.016926	-5.27975	3.13E-11	0.013195	-5.15036	0.000995	0.034437	-4.31635	1.03E-06	0.239893	-1.51282	0.135611	0.350104	-0.95218	0.466004	3.13047	4.56042	5.44E-26
	ENSMUSG00000020473	Aebp1	0.010156	-4.65826	0.000232	0.013195	-3.94276	0.25499	0.026784	-3.42309	0.023995	0.253733	-0.09293	1	0.162839	-0.82308	0.929576	1.182622	3.680559	9.14E-08
	ENSMUSG00000025026	Add3	0.084631	-2.95207	1.67E-08	0.118754	-2.28361	0.024933	0.091831	-2.79079	5.91E-06	0.461333	-0.29866	0.944079	0.460021	-0.31121	0.944929	2.152198	3.162459	1.30E-19
	ENSMUSG00000090019	Gimap1	0.010156	-4.5219	2.08E-08	0.059377	-2.04016	0.205796	0.015305	-3.97598	1.41E-05	0.267573	0.156728	0.948211	0.162839	-0.67107	0.72222	1.004359	3.35518	6.07E-14
	ENSMUSG00000026817	Ak1	0.003385	-5.72555	5.2E-11	0.059377	-2.23974	0.120924	0.076526	-2.04233	0.009437	0.0692	-2.12964	0.016622	0.101775	-1.60607	0.06241	1.413059	4.54167	2.15E-26
	ENSMUSG00000026814	Eng	0.044008	-5.00329	6.85E-16	0.171533	-2.91017	0.006211	0.428544	-1.68285	0.007053	0.226053	-2.59161	0.000204	0.366388	-1.9064	0.002812	6.078328	4.607994	1.48E-38
	ENSMUSG00000027931	Npr1	0.010156	-4.84131	1.03E-11	0.052779	-2.52007	0.03204	0.110962	-1.62422	0.017079	0.046133	-2.81423	0.000297	0.158768	-1.06402	0.217948	1.482625	4.268689	2.00E-29
	ENSMUSG00000027082	Tfpi	0.013541	-4.66969	1.11E-11	0.131949	-1.41551	0.321617	0.149225	-1.33949	0.057832	0.05536	-2.72283	0.000369	0.138413	-1.43962	0.050811	1.604366	4.092711	9.55E-28

ENSMUSG00000074743	Thbd	0.013541	-4.30929	3.66E-09	0.092364	-1.55044	0.347484	0.10331	-1.51149	0.054776	0.059973	-2.24449	0.008239	0.101775	-1.51788	0.068608	1.260884	4.135247	6.88E-24
ENSMUSG00000036867	Smad6	0.044008	-3.39052	2.58E-09	0.191325	-1.11387	0.486401	0.19514	-1.17397	0.08649	0.115333	-1.92164	0.006158	0.158768	-1.47722	0.028122	1.752193	3.69712	3.28E-25
ENSMUSG00000026335	Pam	0.169262	-2.2916	8.42E-05	0.382651	-0.93459	0.778461	0.417065	-0.86854	0.389389	0.272187	-1.49929	0.080354	0.407098	-0.89766	0.409668	2.630464	3.027442	7.59E-14
ENSMUSG00000005973	Rcn1	0.115098	-2.11776	1.32E-05	0.20452	-1.1203	0.409568	0.244882	-0.92647	0.183498	0.184533	-1.33029	0.051534	0.264614	-0.79293	0.348516	1.591322	2.985809	2.22E-18
ENSMUSG00000070436	Serpinh1	0.044008	-4.28684	1.63E-09	0.290287	-1.42345	0.498822	0.294624	-1.49552	0.077006	0.364453	-1.13191	0.311005	0.236117	-1.81785	0.030242	3.308732	3.828874	2.23E-19
ENSMUSG00000008398	Elk3	0.06432	-3.92893	9.95E-14	0.409041	-1.0836	0.41875	0.336713	-1.46502	0.009732	0.53976	-0.69433	0.442603	0.358246	-1.3562	0.024965	3.39569	3.402967	3.95E-25
ENSMUSG00000095098	Ccdc85b	0.074475	-2.83602	3.09E-08	0.191325	-1.30176	0.286003	0.233403	-1.09077	0.094813	0.31832	-0.57137	0.599555	0.219833	-1.17227	0.08192	1.734802	3.103902	1.36E-19
ENSMUSG00000020717	Pecam1	0.020311	-5.79034	1.03E-19	0.705925	-0.60398	0.929203	0.478285	-1.27673	0.037743	0.23528	-2.30779	0.000354	0.33382	-1.81616	0.0022	4.695704	3.881681	7.77E-31
ENSMUSG00000025279	Dnase1l3	0.054164	-5.32639	5.94E-20	1.352473	-0.48172	1	1.289457	-0.59877	0.542435	0.42904	-2.27111	0.000335	0.753131	-1.44701	0.017776	7.500083	3.386822	5.77E-24
ENSMUSG00000028864	Hgf	0.00677	-5.07712	1.9E-11	0.290287	0.134055	1	0.153051	-0.9319	0.297868	0.073813	-1.97632	0.012591	0.113987	-1.37269	0.079248	1.082621	3.275051	5.88E-17
ENSMUSG00000030930	Chst15	0.016926	-4.65698	4.87E-13	0.217715	-0.9468	0.634325	0.271666	-0.67303	0.489605	0.17992	-1.28663	0.090396	0.183194	-1.28385	0.069051	1.634801	3.301197	3.62E-20
ENSMUSG00000029163	Emilin1	0.037238	-4.51628	1.22E-14	0.382651	-1.01653	0.54118	0.642815	-0.24073	0.99813	0.336773	-1.25793	0.080233	0.419311	-0.93653	0.229489	2.704378	2.932291	5.14E-17
ENSMUSG00000041481	Serpina3g	0.057549	-4.6433	4.14E-12	0.963224	-0.36386	1	1.323894	0.159991	0.958991	0.51208	-1.3789	0.128436	0.427453	-1.67904	0.030298	4.217438	2.762191	3.13E-11
ENSMUSG00000024451	Arap3	0.023697	-3.56744	1.57E-09	0.184728	-0.48688	1	0.198967	-0.4236	0.780056	0.050747	-2.42224	0.000589	0.118058	-1.23939	0.073032	1.013055	3.213054	1.41E-19
ENSMUSG00000048200	Cracr2b	0.033852	-3.4119	1.26E-09	0.211118	-0.61805	0.912769	0.225751	-0.57308	0.585603	0.106107	-1.69895	0.012459	0.1262	-1.47168	0.022056	1.265231	3.247112	1.05E-20
ENSMUSG00000045664	Cdc42ep2	0.037238	-3.31901	1.58E-08	0.244105	-0.43523	1	0.198967	-0.81747	0.353072	0.170693	-1.02574	0.23456	0.146555	-1.28288	0.078624	1.234796	3.054564	1.95E-16
ENSMUSG00000030123	Plxnd1	0.132025	-3.18199	6.33E-11	0.844471	-0.26031	1	0.604553	-0.83864	0.227889	0.45672	-1.25087	0.057787	0.45595	-1.27524	0.034815	3.726128	3.018745	4.38E-20
ENSMUSG00000019970	Sgk1	0.318213	-3.01077	9.68E-11	1.860475	-0.20345	1	1.32772	-0.78418	0.268631	0.862693	-1.43446	0.020381	0.972964	-1.27002	0.031074	7.83487	2.991455	9.86E-15
ENSMUSG00000022799	Arhgap31	0.010156	-5.61948	2.55E-16	0.158338	-1.79938	0.098382	0.156878	-1.93068	0.001395	0.295253	-0.92808	0.271449	0.317537	-0.83159	0.348435	2.286982	3.663249	1.11E-25
ENSMUSG00000042286	Stab1	0.104943	-4.04162	4.4E-15	0.824678	-0.85934	0.631013	0.642815	-1.31681	0.021167	1.300959	-0.15617	1	0.9119	-0.75025	0.365689	5.073969	2.833809	1.63E-17
ENSMUSG00000022816	Fstl1	0.003385	-6.08283	1.83E-06	0.171533	-1.12049	1	0.10331	-1.97959	0.203528	0.216827	-0.81652	0.893183	0.187265	-1.06527	0.745296	1.560887	3.625089	1.52E-08
ENSMUSG00000022665	Ccdc80	0.00677	-5.7699	6.77E-11	0.277092	-0.69656	1	0.183662	-1.40745	0.141726	0.253733	-0.87276	0.563307	0.252401	-0.89987	0.548624	1.752193	3.286655	8.02E-13
ENSMUSG00000027669	Gnb4	0.010156	-4.8975	1.93E-13	0.217715	-0.57498	0.954291	0.191314	-0.84426	0.284614	0.212213	-0.65245	0.524579	0.154697	-1.16532	0.099619	1.204361	3.043778	5.86E-18
ENSMUSG00000004837	Grap	0.023697	-3.71822	4.68E-10	0.211118	-0.44465	1	0.168356	-0.86394	0.27775	0.19376	-0.61354	0.577437	0.175052	-0.79159	0.389787	1.004359	2.799791	8.28E-15
ENSMUSG00000026043	Col3a1	0.067705	-4.791	0.000348	0.395846	-2.09138	0.913989	0.726994	-1.26841	0.733863	1.462426	-0.11424	1	1.131733	-0.54798	1	5.756586	2.956243	1.07E-04
ENSMUSG00000086320	Gm12840	0.047393	-4.82685	2.19E-10	0.46182	-1.39788	0.570722	0.635163	-0.9852	0.407701	1.250213	0.152094	0.933873	0.553653	-1.19186	0.29617	4.108741	2.866442	5.97E-10
ENSMUSG00000022122	Ednrb	0.013541	-5.48354	2.37E-12	0.112156	-2.47855	0.071926	0.141572	-2.27959	0.002418	0.715066	0.31156	0.759256	0.606576	0.027128	1	2.069588	2.736709	3.41E-10
ENSMUSG00000019880	Rspo3	0.003385	-5.38061	0.015055	0.059377	-1.88673	1	0.114788	-1.06541	1	0	-5.84139	0.14576	0.036639	-2.69248	0.617601	1.139143	4.737752	1.60E-06
ENSMUSG00000023078	Cxcl13	0.050779	-4.40019	0.17175	0.712522	-0.38712	1	0.501243	-0.9968	1	0.032293	-4.86547	0.640009	0.956681	0.087651	0.871549	3.386994	2.992917	1.35E-02
ENSMUSG00000035459	Stab2	0.091402	-4.0557	6.34E-15	1.365667	0.12948	1	0.948918	-0.48706	0.663889	0.119947	-3.56514	6.27E-08	0.801983	-0.75659	0.363696	4.604399	2.929013	1.29E-18
