# Supplementary material for: Single-Cell Transcriptomes Reveal Characteristic Features of Mouse Hepatocytes with Liver Cholestatic Injury
Source: Cells. 2019 Sep 11;8(9):1069. doi: 10.3390/cells8091069 (PMC6770527; doi:10.3390/cells8091069)
Supplement: Supplementary file 1 [file cells-08-01069-s001.zip › supplemental figures and tables/Supplemental Table 4.pdf]

Single-cell transcriptomes reveal characteristic features of Mouse Hepatocytes with Cholestasis Injury

Na Chang, Lei Tian, Xiaofang Ji, Xuan Zhou, Lei Hou, Xinhao Zhao, Yuanru Yang, Lin Yang, Liying Li

Supplemental Table 4: High expressed genes of BDL-2-5 (Related to Figure 6-7)

Top 30 highly expressed genes in BDL-2, BDL-3, and BDL-5:

EnsemblID	GeneName	Cluster 1 Average	Cluster 1 Log2 Fold Change	Cluster 1 P-Value	Cluster 2 Average	Cluster 2 Log2 Fold Change	Cluster 2 P-Value	Cluster 3 Average	Cluster 3 Log2 Fold Change	Cluster 3 P-Value	Cluster 4 Average	Cluster 4 Log2 Fold Change	Cluster 4 P-Value	Cluster 5 Average	Cluster 5 Log2 Fold Change	Cluster 5 P-Value	Cluster 6 Average	Cluster 6 Log2 Fold Change	Cluster 6 P-Value
ENSMUSG00000098188	Sowahc	0.13	-2.48	2.19E-07	2.01	2.14	7.57E-05	0.91	0.73	1.51E-01	0.29	-1.21	7.26E-02	0.6	-0.06	1.00E+00	0.33	-1	1.12E-01
ENSMUSG00000040724	Kcna2	0.02	-5.17	4.94E-17	1.83	2.1	7.57E-05	1.23	1.52	1.03E-04	0.06	-3.5	3.65E-07	0.56	-0.05	1.00E+00	0.25	-1.36	2.17E-02
ENSMUSG00000005413	Hmox1	0.61	-3.92	7.48E-15	22.77	2.04	1.51E-04	10.99	0.75	1.63E-01	3.1	-1.4	3.38E-02	8.96	0.35	7.46E-01	4.1	-0.97	1.07E-01
ENSMUSG00000060044	Tmem26	0.03	-4.02	1.24E-12	1.35	2.03	1.20E-04	0.83	1.24	2.80E-03	0.05	-3.34	1.77E-06	0.55	0.4	6.37E-01	0.18	-1.43	1.69E-02
ENSMUSG00000032915	Adgre4	0.07	-4.39	9.34E-18	3.89	1.97	7.57E-05	1.9	0.7	1.47E-01	0.44	-1.73	2.91E-03	1.93	0.71	1.49E-01	0.65	-1.13	4.70E-02
ENSMUSG00000035448	Ccr3	0.02	-4.55	3.15E-11	1.12	1.96	1.34E-03	0.73	1.32	5.99E-03	0.11	-1.95	1.19E-02	0.44	0.26	8.62E-01	0.13	-1.62	2.30E-02
ENSMUSG00000022181	C6	1.02	-1.95	2.37E-06	9.8	1.95	7.57E-05	5.87	1.1	4.65E-03	0.41	-3.23	1.85E-08	3.48	0.08	1.00E+00	1.63	-1.16	2.92E-02
ENSMUSG00000064225	Paqr9	0.35	-2.1	8.44E-07	3.7	1.93	7.57E-05	2.1	0.97	1.62E-02	0.23	-2.66	4.30E-06	1.59	0.41	5.50E-01	0.51	-1.47	5.47E-03
ENSMUSG00000044206	Vsig4	0.62	-4.53	2.61E-22	32.97	1.88	1.23E-04	22.88	1.36	1.85E-04	1.79	-2.89	1.98E-07	14.23	0.38	6.35E-01	4.52	-1.51	1.66E-03
ENSMUSG00000022103	Gfra2	0.02	-4.38	6.42E-14	1.27	1.86	6.12E-04	0.8	1.13	7.61E-03	0.04	-3.66	3.07E-07	0.68	0.76	1.51E-01	0.19	-1.36	2.43E-02
ENSMUSG00000015354	Pcolce2	0.04	-4.61	8.26E-17	2.24	1.86	3.44E-04	1.55	1.32	6.82E-04	0.06	-3.84	1.22E-08	1.09	0.58	3.31E-01	0.31	-1.48	8.51E-03
ENSMUSG00000025743	Sdc3	0.65	-4.65	1.05E-23	36.5	1.82	1.94E-04	20.57	0.85	4.39E-02	4.81	-1.61	1.75E-03	17.5	0.52	3.90E-01	8.27	-0.77	1.57E-01
ENSMUSG00000028917	Plekhm2	0.16	-2.42	1.43E-07	1.87	1.74	9.36E-04	1.09	0.83	6.80E-02	0.45	-0.73	3.73E-01	0.65	-0.13	1.00E+00	0.48	-0.62	3.97E-01
ENSMUSG00000030830	Itgal	0.17	-4.1	2.54E-18	6.34	1.74	6.12E-04	3.84	0.92	2.84E-02	1.17	-1.14	5.75E-02	3.19	0.54	3.36E-01	1.15	-1.18	2.61E-02
ENSMUSG00000026390	Marco	0.12	-4.76	5.23E-12	7.09	1.7	2.37E-02	3.24	0.33	7.76E-01	1.2	-1.31	1.92E-01	4.8	1.08	8.60E-02	1.69	-0.78	4.77E-01
ENSMUSG00000004359	Spic	0.02	-4.78	5.01E-15	1.14	1.68	2.44E-03	0.76	1.05	1.61E-02	0.17	-1.49	2.34E-02	0.59	0.52	4.56E-01	0.26	-0.85	2.11E-01
ENSMUSG00000063193	Cd300lb	0.02	-4.59	4.56E-15	1.16	1.65	2.23E-03	0.74	0.94	3.43E-02	0.53	0.27	7.59E-01	0.42	-0.13	1.00E+00	0.18	-1.45	1.26E-02
ENSMUSG00000013089	Etv5	0.07	-2.85	4.26E-09	1.15	1.63	2.30E-03	0.62	0.56	3.25E-01	0.21	-1.22	6.03E-02	0.63	0.58	3.34E-01	0.35	-0.43	6.42E-01
ENSMUSG00000070873	Lilra5	0.04	-4.78	1.03E-18	2.21	1.6	2.17E-03	1.79	1.4	1.67E-04	0.47	-1.02	1.21E-01	1.01	0.24	8.30E-01	0.33	-1.58	3.12E-03
ENSMUSG00000034641	Cd300ld	0.1	-4.59	1.40E-20	4.92	1.56	2.17E-03	4.05	1.39	1.56E-04	0.94	-1.23	3.84E-02	2.36	0.29	7.46E-01	0.85	-1.39	7.47E-03
ENSMUSG00000014542	Clec4f	1.24	-4.37	2.54E-21	49.89	1.54	3.02E-03	45.98	1.64	4.39E-06	3.17	-2.91	1.81E-07	25.66	0.39	6.18E-01	8.19	-1.5	1.98E-03
ENSMUSG00000072596	Ear2	0.05	-4.15	2.35E-14	1.66	1.33	2.43E-02	1.56	1.42	3.07E-04	0.43	-0.92	2.31E-01	0.96	0.4	6.11E-01	0.3	-1.52	8.90E-03
ENSMUSG00000070427	Il18bp	0.36	-3.67	2.26E-16	7.98	1.3	1.81E-02	8.03	1.54	1.54E-05	1.68	-1.29	2.41E-02	4.37	0.28	8.02E-01	1.63	-1.35	8.65E-03
ENSMUSG00000074874	Ctla2b	0.11	-4.31	6.50E-19	3.78	1.25	2.20E-02	3.77	1.46	6.73E-05	0.77	-1.38	1.64E-02	1.82	0	1.00E+00	1.48	-0.35	7.23E-01
ENSMUSG00000025877	Hk3	0.02	-5.13	4.97E-18	1.04	1.05	9.13E-02	1.31	1.75	1.69E-06	0.41	-0.53	5.92E-01	0.6	0.11	9.88E-01	0.2	-1.64	2.77E-03
ENSMUSG00000032725	Folr2	0.22	-4.53	3.26E-21	7.92	1.08	8.01E-02	9.66	1.72	1.53E-06	1.4	-1.74	1.63E-03	5.65	0.54	3.80E-01	1.63	-1.52	3.03E-03
ENSMUSG00000030159	Clec1b	0.1	-4.8	5.29E-22	3.98	0.96	1.13E-01	5.12	1.68	2.46E-06	0.65	-1.96	3.60E-04	3.07	0.56	3.09E-01	1.26	-0.96	8.77E-02
ENSMUSG00000017754	Pltp	0.29	-4.68	7.47E-23	11.35	1.06	8.42E-02	13.03	1.54	1.71E-05	4.28	-0.58	4.33E-01	6.57	0.14	9.88E-01	3.23	-1.04	5.64E-02
ENSMUSG00000030703	Gdpd3	0.04	-4.4	4.95E-15	1.11	0.82	2.94E-01	1.45	1.53	8.49E-05	0.87	0.43	5.15E-01	0.56	-0.32	9.10E-01	0.29	-1.36	2.11E-02
ENSMUSG00000019874	Fabp7	0.26	-4.63	5.81E-21	9.63	1.07	9.37E-02	10.86	1.51	4.87E-05	1.51	-1.94	6.37E-04	8.16	0.86	6.29E-02	2.01	-1.51	4.38E-03

specific and high expressed genes in BDL-5:

EnsemblID	GeneName	Cluster 1 Average	Cluster 1 Log2 Fold Change	Cluster 1 P-Value	Cluster 2 Average	Cluster 2 Log2 Fold Change	Cluster 2 P-Value	Cluster 3 Average	Cluster 3 Log2 Fold Change	Cluster 3 P-Value	Cluster 4 Average	Cluster 4 Log2 Fold Change	Cluster 4 P-Value	Cluster 5 Average	Cluster 5 Log2 Fold Change	Cluster 5 P-Value	Cluster 6 Average	Cluster 6 Log2 Fold Change	Cluster 6 P-Value
ENSMUSG00000024681	Ms4a3	0.010156	-4.24959	3.58E-01	0.006597	-4.12535	1.00E+00	0	-6.04496	5.03E-01	0.004613	-4.71586	9.50E-01	1.139875	7.555834	6.37E-08	0.004348	-4.81718	7.63E-01
ENSMUSG00000020125	Elane	0.060934	-5.15226	5.62E-01	0.118754	-4.01791	1.00E+00	0.053568	-5.27581	1.00E+00	0.04152	-5.53955	1.00E+00	10.06754	7.435303	7.85E-07	0.030435	-5.96394	1.00E+00
ENSMUSG00000056399	Prss34	0.016926	-5.35093	9.79E-01	0.046182	-3.79837	1.00E+00	0.022958	-4.90717	1.00E+00	0.009227	-5.81181	1.00E+00	3.639457	7.39065	1.92E-02	0.017391	-5.17301	9.85E-01
ENSMUSG00000040314	Ctsg	0.013541	-5.54278	1.83E-01	0.02639	-4.40842	1.00E+00	0.026784	-4.63995	6.71E-01	0.02768	-4.51019	9.44E-01	3.456263	7.316225	3.11E-08	0.013044	-5.42384	6.65E-01
ENSMUSG00000009350	Mpo	0.040623	-4.99745	2.29E-01	0.065974	-4.10596	1.00E+00	0.045915	-4.77767	6.36E-01	0.04152	-4.8349	9.05E-01	6.134968	7.005823	6.88E-08	0.047827	-4.67133	7.37E-01
ENSMUSG00000022157	Mcpt8	0.033852	-3.70919	9.54E-01	0.019792	-4.05294	1.00E+00	0.015305	-4.64294	1.00E+00	0.101493	-2.0686	9.22E-01	2.047703	5.746611	2.89E-02	0.017391	-4.42019	1.00E+00
ENSMUSG00000045328	Cenpe	0	-6.19787	5.60E-11	0.013195	-3.46798	1.32E-02	0.011479	-3.96221	7.00E-06	0.02768	-2.81472	1.62E-03	1.009603	5.488324	8.37E-35	0.06087	-1.77279	2.60E-02
ENSMUSG00000027306	Nusap1	0.020311	-3.9507	1.05E-08	0.02639	-3.30889	1.13E-02	0.019131	-3.95687	1.63E-06	0.004613	-5.23195	5.18E-07	1.506263	5.44211	1.31E-37	0.100001	-1.66854	2.87E-02
ENSMUSG00000020914	Top2a	0.07109	-3.64322	1.01E-09	0.112156	-2.80398	1.46E-02	0.068873	-3.63916	3.70E-07	0.036907	-4.40704	1.61E-07	3.806367	5.291772	8.61E-43	0.20435	-2.03813	2.77E-03
ENSMUSG00000027715	Ccna2	0.044008	-3.06067	1.32E-06	0.052779	-2.58019	4.39E-02	0.03061	-3.49477	7.64E-06	0.009227	-4.77516	1.30E-06	1.628392	5.261511	2.30E-36	0.073914	-2.24227	2.77E-03
ENSMUSG00000041431	Ccnb1	0.027082	-3.06434	1.29E-05	0.032987	-2.53026	8.64E-02	0.022958	-3.21736	1.57E-04	0.018453	-3.38888	4.55E-04	1.046242	5.23999	1.87E-30	0.034783	-2.62181	1.92E-03
ENSMUSG00000038943	Prc1	0.013541	-4.32094	4.84E-09	0.006597	-4.52007	2.23E-03	0.026784	-3.41144	2.13E-05	0.009227	-4.52177	6.19E-06	1.347495	5.08024	1.73E-32	0.134784	-1.09194	2.19E-01
ENSMUSG00000001403	Ube2c	0.06432	-3.90977	8.58E-09	0.178131	-2.27966	1.05E-01	0.114788	-3.0435	7.26E-05	0.073813	-3.60424	5.63E-05	4.070981	5.056437	9.23E-33	0.208698	-2.13668	6.77E-03
ENSMUSG00000094777	Hist1h2ap	0.054164	-4.80354	1.92E-11	0.131949	-3.36107	1.23E-02	0.172183	-3.12385	4.78E-05	0.239893	-2.58962	2.59E-03	6.367014	5.033354	1.06E-32	0.391309	-1.87914	1.93E-02
ENSMUSG00000040204	2810417H13	0.037238	-4.50356	3.21E-12	0.105559	-2.85958	1.35E-02	0.091831	-3.20631	4.35E-06	0.133787	-2.612	5.57E-04	3.643528	4.991723	3.61E-38	0.221742	-1.88761	6.59E-03
ENSMUSG00000020649	Rrm2	0.060934	-2.7618	3.62E-05	0.059377	-2.58019	7.78E-02	0.019131	-4.24346	2.17E-06	0.018453	-4.18535	2.80E-05	1.762735	4.945919	4.24E-29	0.126088	-1.62694	5.74E-02
ENSMUSG00000017716	Birc5	0.101557	-3.16523	2.95E-08	0.145143	-2.47259	2.97E-02	0.065047	-3.74768	1.56E-07	0.08304	-3.34424	1.39E-05	3.733089	4.832708	1.46E-37	0.273916	-1.63029	1.81E-02
ENSMUSG00000027469	Tpx2	0.016926	-3.84579	2.05E-08	0.039585	-2.48455	5.00E-02	0.045915	-2.47883	5.91E-04	0.04152	-2.54694	1.87E-03	1.127662	4.703847	9.02E-31	0.073914	-1.76351	1.72E-02
ENSMUSG00000031004	Mki67	0.030467	-4.15078	2.66E-11	0.184728	-1.43842	2.96E-01	0.091831	-2.57605	7.47E-05	0.064587	-3.01104	6.14E-05	2.28382	4.568773	1.08E-34	0.152176	-1.80254	7.04E-03
ENSMUSG00000026605	Cenpf	0.037238	-2.90289	1.73E-05	0.046182	-2.37062	1.02E-01	0.038263	-2.81294	4.89E-04	0.023067	-3.38466	3.65E-04	1.172442	4.545707	2.63E-25	0.104349	-1.34437	1.24E-01
ENSMUSG00000023505	Cdca3	0.050779	-2.76162	1.75E-06	0.046182	-2.65548	2.43E-02	0.034437	-3.24112	6.86E-06	0.050747	-2.64698	5.63E-04	1.420772	4.530226	3.50E-32	0.12174	-1.41404	4.58E-02
ENSMUSG00000023015	Racgap1	0.016926	-3.8812	9.24E-09	0.052779	-2.1487	8.68E-02	0.03061	-3.06328	3.49E-05	0.0692	-1.87792	1.81E-02	1.111378	4.372598	2.74E-28	0.108697	-1.233	1.09E-01
ENSMUSG00000057729	Prtn3	0.196344	-3.34196	4.31E-02	0.138546	-3.65453	7.92E-01	0.145399	-3.73229	1.08E-01	0.94112	-0.90372	1.00E+00	7.559811	4.326746	5.30E-07	0.486962	-1.92658	5.73E-01
ENSMUSG00000001228	Uhrf1	0.044008	-2.64121	1.48E-05	0.079169	-1.61819	2.63E-01	0.015305	-3.94615	1.67E-06	0.032293	-2.93103	4.26E-04	1.115449	4.309682	7.16E-27	0.12174	-1.0801	1.90E-01
ENSMUSG00000028873	Cdca8	0.033852	-3.09573	1.77E-07	0.046182	-2.43309	3.41E-02	0.034437	-3.01756	1.71E-05	0.0692	-1.99062	7.76E-03	1.184655	4.289304	7.26E-30	0.12174	-1.17871	1.09E-01
ENSMUSG00000019942	Cdk1	0.067705	-2.86739	3.54E-07	0.112156	-1.96297	9.36E-02	0.080352	-2.57797	8.95E-05	0.046133	-3.28143	2.22E-05	1.901148	4.133343	1.13E-28	0.243481	-0.89051	2.73E-01
ENSMUSG00000032254	Kif23	0.010156	-4.60763	8.50E-11	0.138546	-0.94068	7.09E-01	0.072699	-2.00107	3.33E-03	0.036907	-2.87391	3.21E-04	1.176513	4.123269	1.13E-26	0.113045	-1.31605	7.52E-02
ENSMUSG00000024590	Lmnb1	0.06432	-2.59809	1.92E-06	0.079169	-2.10739	5.97E-02	0.076526	-2.30472	3.16E-04	0.147627	-1.28967	9.54E-02	1.428914	3.781303	3.07E-25	0.156523	-1.21273	8.08E-02
ENSMUSG00000032218	Ccnb2	0.06432	-2.29755	8.33E-05	0.046182	-2.53153	3.84E-02	0.118615	-1.35173	7.21E-02	0.073813	-1.99991	1.19E-02	1.164301	3.749813	1.80E-21	0.113045	-1.39428	6.33E-02
ENSMUSG00000054717	Hmgb2	0.192959	-4.00963	4.09E-13	0.930237	-1.54832	2.01E-01	0.658121	-2.16482	3.16E-04	1.591599	-0.76477	4.49E-01	10.49092	3.643427	2.93E-14	1.091316	-1.36522	3.76E-02

Top 30 highly expressed genes in BDL-4:

EnsemblID	GeneName	Cluster 1 Average	Cluster 1 Log2 Fold Change	Cluster 1 P-Value	Cluster 2 Average	Cluster 2 Log2 Fold Change	Cluster 2 P-Value	Cluster 3 Average	Cluster 3 Log2 Fold Change	Cluster 3 P-Value	Cluster 4 Average	Cluster 4 Log2 Fold Change	Cluster 4 P-Value	Cluster 5 Average	Cluster 5 Log2 Fold Change	Cluster 5 P-Value	Cluster 6 Average	Cluster 6 Log2 Fold Change	Cluster 6 P-Value
ENSMUSG00000029379	Cxcl3	0.023697	-4.9702	1.00E+00	0.02639	-4.51853	1.00E+00	0.053568	-3.8327	1.00E+00	4.179678	7.059515	1.51E-03	0.008142	-6.06433	1.00E+00	0.039131	-4.20266	1.00E+00
ENSMUSG00000009185	Ccl8	0.003385	-6.07998	6.07E-02	0.032987	-3.34918	1.00E+00	0.198967	-0.97869	1.00E+00	1.882239	4.505257	3.10E-04	0.113987	-1.81412	1.00E+00	0.047827	-3.02363	8.00E-01

ENSMUSG00000035042	Ccl5	0.047393	-5.55105	1.85E-03	0.145143	-3.79562	8.26E-01	1.105796	-0.89859	1.00E+00	8.193276	3.486021	2.46E-04	1.893006	0.006812	9.77E-01	0.313047	-2.79335	3.47E-01
ENSMUSG00000058427	Cxcl2	0.084631	-3.58551	8.64E-01	0.197923	-2.19119	1.00E+00	0.505069	-0.8719	9.74E-01	3.584558	3.357638	3.88E-02	0.411169	-1.1876	1.00E+00	0.539136	-0.74952	8.45E-01
ENSMUSG00000022584	Ly6c2	0.084631	-3.36807	1.37E-04	0.085767	-3.14397	1.46E-01	0.210446	-1.99751	8.61E-02	3.012505	3.250262	9.05E-08	1.062526	0.680985	5.30E-01	0.08261	-3.31266	3.27E-03
ENSMUSG00000024672	Ms4a7	0.027082	-4.63382	4.04E-11	0.244105	-1.3704	4.59E-01	0.497417	-0.32237	9.78E-01	2.412772	3.16002	1.53E-12	0.398956	-0.68104	6.54E-01	0.195654	-1.77218	2.02E-02
ENSMUSG00000040751	Lat2	0.013541	-4.7292	1.08E-12	0.092364	-1.98043	7.67E-02	0.156878	-1.32694	4.61E-02	1.416293	3.125834	7.02E-15	0.33382	-0.1001	1.00E+00	0.217394	-0.79546	3.25E-01
ENSMUSG00000002204	Napsa	0.003385	-5.85473	6.04E-12	0.072572	-2.10162	1.27E-01	0.156878	-1.10245	2.31E-01	1.213306	3.079013	1.68E-11	0.394885	0.451155	6.66E-01	0.08261	-2.02887	8.59E-03
ENSMUSG00000049103	Ccr2	0.067705	-4.3167	2.19E-13	0.349664	-1.78897	1.19E-01	0.89535	-0.40015	8.47E-01	4.419571	3.063995	2.30E-14	1.005532	-0.19967	1.00E+00	0.300003	-2.09122	1.02E-03
ENSMUSG00000030144	Clec4d	0.030467	-3.45387	1.22E-04	0.079169	-1.93038	5.28E-01	0.149225	-1.12418	4.97E-01	1.125653	2.937704	1.65E-06	0.236117	-0.38045	1.00E+00	0.239133	-0.35378	1.00E+00
ENSMUSG00000036594	H2-Aa	0.802303	-5.10867	4.07E-21	11.59828	-1.01516	4.09E-01	20.61219	-0.12279	1.00E+00	83.28446	2.936245	4.87E-13	16.62181	-0.48569	6.66E-01	5.95224	-2.08671	1.28E-04
ENSMUSG00000060586	H2-Eb1	0.646582	-4.4857	1.03E-16	6.84813	-0.83714	6.08E-01	10.57585	-0.16585	1.00E+00	43.54984	2.915931	4.87E-13	7.852922	-0.66239	4.67E-01	3.843521	-1.77495	3.14E-03
ENSMUSG00000029816	Gpnmb	0.091402	-4.53447	3.92E-08	0.71912	-1.37641	7.30E-01	2.020277	0.259425	8.72E-01	6.55093	2.909674	4.13E-07	0.858977	-1.17131	4.27E-01	0.665225	-1.55543	1.52E-01
ENSMUSG00000021451	Sema4d	0.044008	-2.8924	2.64E-07	0.145143	-1.00797	5.76E-01	0.206619	-0.53749	6.55E-01	1.065679	2.8926	4.87E-13	0.211691	-0.4904	7.54E-01	0.12174	-1.34236	4.77E-02
ENSMUSG00000069515	Lyz1	0	-7.04821	1.02E-09	0.052779	-2.72037	1.86E-01	0.19514	-0.97078	5.63E-01	1.310186	2.884583	1.05E-06	0.227975	-0.71313	8.17E-01	0.4087	0.268689	7.66E-01
ENSMUSG00000024610	Cd74	5.216662	-3.59232	5.62E-14	28.00608	-0.93934	4.68E-01	46.0761	-0.1706	1.00E+00	186.5124	2.849114	4.87E-13	38.99593	-0.45096	7.04E-01	13.58276	-2.09746	9.11E-05
ENSMUSG00000073421	H2-Ab1	1.357483	-3.95998	2.81E-15	11.84238	-0.5773	8.52E-01	15.92116	-0.1149	1.00E+00	59.86258	2.712145	4.87E-13	13.32839	-0.41575	7.46E-01	4.973968	-1.96072	2.64E-04
ENSMUSG00000021728	Emb	0.023697	-3.95782	9.97E-10	0.131949	-1.39015	3.60E-01	0.187488	-0.9573	2.71E-01	1.176399	2.689123	5.45E-10	0.476305	0.6438	3.68E-01	0.095653	-1.94432	5.65E-03
ENSMUSG00000025804	Ccr1	0.037238	-3.75805	1.07E-09	0.197923	-1.2085	4.83E-01	0.248708	-0.94419	2.70E-01	1.517786	2.640407	6.88E-10	0.49666	0.219992	9.02E-01	0.256525	-0.87454	2.96E-01
ENSMUSG00000069516	Lyz2	1.926204	-4.80084	5.25E-21	18.53877	-1.30886	1.68E-01	35.26302	-0.33842	8.09E-01	144.7571	2.583151	2.73E-12	39.19133	-0.15491	1.00E+00	27.26552	-0.75257	2.34E-01
ENSMUSG00000099974	Bcl2a1d	0.010156	-5.63706	1.01E-11	0.283689	-0.96343	8.51E-01	0.554811	0.078546	1.00E+00	1.799199	2.579301	2.71E-07	0.464092	-0.22953	1.00E+00	0.217394	-1.42783	1.09E-01
ENSMUSG00000074677	Sirpb1c	0.016926	-4.86139	3.04E-14	0.303482	-0.66356	8.84E-01	0.478285	0.044655	1.00E+00	1.536239	2.492324	5.64E-10	0.443737	-0.08528	1.00E+00	0.160871	-1.68318	7.04E-03
ENSMUSG00000027533	Fabp5	0.473934	-2.34399	7.79E-04	0.395846	-2.46101	1.40E-01	1.040749	-1.09791	3.58E-01	6.504797	2.453242	9.89E-06	0.761273	-1.57475	1.26E-01	3.226123	0.864493	1.84E-01
ENSMUSG00000015437	Gzmb	0.013541	-4.80305	1.59E-02	0.019792	-3.99339	9.22E-01	0.007653	-5.32576	5.29E-02	1.199466	2.422067	2.77E-02	1.029958	2.129984	5.78E-02	0.008696	-5.10302	8.86E-02
ENSMUSG00000037548	H2-DMb2	0.037238	-4.26809	2.08E-13	0.422235	-0.60489	9.37E-01	0.654294	0.088827	1.00E+00	1.983732	2.401138	1.55E-09	0.659499	0.101757	1.00E+00	0.152176	-2.19991	2.74E-04
ENSMUSG00000056290	Ms4a4b	0.016926	-4.65698	8.87E-10	0.263897	-0.65778	1.00E+00	0.348192	-0.26038	1.00E+00	1.277893	2.368194	2.03E-06	0.517015	0.437599	6.84E-01	0.126088	-1.8299	2.54E-02
ENSMUSG00000026879	Gsn	0.013541	-4.75805	7.72E-13	0.250702	-0.55966	9.77E-01	0.309929	-0.26397	9.78E-01	1.134879	2.353458	1.55E-08	0.33382	-0.13429	1.00E+00	0.247829	-0.61951	4.85E-01
ENSMUSG00000029373	Pf4	0.037238	-5.00272	2.46E-02	0.389248	-1.49738	1.00E+00	0.711689	-0.63352	1.00E+00	3.215492	2.340346	4.62E-02	0.858977	-0.31478	1.00E+00	1.226101	0.301233	6.89E-01
ENSMUSG00000023992	Trem2	0.047393	-5.05248	4.46E-18	0.606963	-1.22398	3.43E-01	1.453987	0.150558	9.54E-01	4.133545	2.322237	3.50E-09	1.302714	-0.04472	1.00E+00	0.734791	-0.98178	1.43E-01
ENSMUSG00000000682	Cd52	0.284361	-4.69695	3.96E-21	3.879287	-0.67868	7.98E-01	7.044187	0.307004	7.55E-01	18.03812	2.273061	6.79E-10	6.383298	0.124789	1.00E+00	1.395668	-2.29073	1.16E-05
