# Supplementary material for: Single-Cell Transcriptomes Reveal Characteristic Features of Mouse Hepatocytes with Liver Cholestatic Injury
Source: Cells. 2019 Sep 11;8(9):1069. doi: 10.3390/cells8091069 (PMC6770527; doi:10.3390/cells8091069)
Supplement: Supplementary file 1 [file cells-08-01069-s001.zip › supplemental figures and tables/Supplemental Table 5.pdf]

Single-cell transcriptomes reveal characteristic features of Mouse Hepatocytes with Cholestasis Injury

Na Chang, Lei Tian, Xiaofang Ji, Xuan Zhou, Lei Hou, Xinhao Zhao, Yuanru Yang, Lin Yang, Liying Li

Supplemental Table 5: High expressed genes of BDL-1 (Related to Supplementary Figure 2)

EnsemblID	GeneName	Cluster 1 Average	Cluster 1 Log2 Fold Change	Cluster 1 P-Value	Cluster 2 Average	Cluster 2 Log2 Fold Change	Cluster 2 P-Value	Cluster 3 Average	Cluster 3 Log2 Fold Change	Cluster 3 P-Value	Cluster 4 Average	Cluster 4 Log2 Fold Change	Cluster 4 P-Value	Cluster 5 Average	Cluster 5 Log2 Fold Change	Cluster 5 P-Value	Cluster 6 Average	Cluster 6 Log2 Fold Change	Cluster 6 P-Value
ENSMUSG00000032068	Plet1	1.448885	6.323067	1.26E-35	0.013195	-4.18142	1.32E-02	0.022958	-3.8606	4.99E-05	0.018453	-4.02848	2.34E-04	0.020355	-3.97744	5.01E-05	0.008696	-4.87325	4.08E-06
ENSMUSG00000031271	Serpina7	1.922819	6.297559	9.47E-42	0.032987	-3.58509	1.35E-02	0.022958	-4.27483	1.88E-06	0.036907	-3.58328	1.65E-04	0	-6.98798	7.07E-10	0.030435	-3.85698	1.16E-05
ENSMUSG00000044393	Dsg2	1.92959	5.73558	5.52E-49	0.039585	-3.39653	3.40E-03	0.026784	-4.11607	5.03E-08	0.018453	-4.47724	1.82E-07	0.044781	-3.41269	1.13E-06	0.047827	-3.29876	2.58E-06
ENSMUSG00000030237	Slco1a4	2.024376	5.571985	2.33E-35	0.098961	-2.26469	1.49E-01	0.042089	-3.60343	2.44E-05	0.036907	-3.70121	1.36E-04	0.024426	-4.28355	2.20E-06	0.026087	-4.16963	4.31E-06
ENSMUSG00000000567	Sox9	3.331081	5.38471	6.25E-49	0.131949	-2.61208	1.57E-02	0.072699	-3.59842	1.42E-07	0.0692	-3.60175	1.69E-06	0.069207	-3.64434	5.88E-08	0.069566	-3.61424	1.03E-07
ENSMUSG00000010175	Prox1	1.844958	5.378209	1.86E-48	0.02639	-3.84812	7.69E-04	0.049742	-3.2551	1.06E-06	0.02768	-3.94831	4.97E-07	0.048852	-3.25567	9.60E-07	0.056522	-3.03235	4.07E-06
ENSMUSG00000020926	Adam11	1.062967	5.245173	2.63E-33	0.02639	-3.05718	2.97E-02	0.015305	-3.97176	6.57E-06	0.04152	-2.62638	3.07E-03	0.008142	-4.60834	8.79E-07	0.047827	-2.45605	2.52E-03
ENSMUSG00000021336	Slc17a4	1.611376	5.213044	5.71E-37	0.046182	-2.9795	2.06E-02	0.061221	-2.78128	1.67E-04	0.046133	-3.10218	2.53E-04	0.036639	-3.45774	1.03E-05	0.021739	-4.09194	7.98E-07
ENSMUSG00000031640	Klkb1	1.31686	5.185563	1.27E-41	0.072572	-2.08788	7.25E-02	0.038263	-3.13143	8.45E-06	0.023067	-3.69895	6.53E-06	0.024426	-3.6884	5.67E-07	0.030435	-3.37841	2.98E-06
ENSMUSG00000078650	G6pc	2.853761	5.178317	9.83E-36	0.151741	-2.20548	1.17E-01	0.088005	-3.12005	4.86E-05	0.059973	-3.58941	5.63E-05	0.061065	-3.60887	6.64E-06	0.052174	-3.79922	3.18E-06
ENSMUSG00000045991	Onecut2	2.342589	5.166948	4.19E-41	0.112156	-2.34052	4.96E-02	0.057394	-3.42888	1.98E-06	0.04152	-3.79439	4.30E-06	0.097704	-2.65876	1.33E-04	0.026087	-4.41601	1.50E-08
ENSMUSG00000025194	Abcc2	1.299934	5.166948	2.26E-36	0.052779	-2.496	5.25E-02	0.045915	-2.86612	1.13E-04	0.023067	-3.68183	3.78E-05	0.020355	-3.89708	1.67E-06	0.039131	-3.03235	7.92E-05
ENSMUSG00000027801	Tm4sf4	16.20855	5.097764	2.46E-50	0.567379	-2.87348	4.20E-03	0.428544	-3.40362	4.86E-08	0.309093	-3.8247	4.88E-08	0.744989	-2.57157	1.74E-05	0.317395	-3.80238	1.29E-09
ENSMUSG00000035186	Ubd	1.323631	5.055439	1.94E-24	0.039585	-2.90296	1.14E-01	0.049742	-2.7938	3.60E-03	0.036907	-3.12416	4.75E-03	0.04071	-3.04313	2.53E-03	0.026087	-3.59479	4.64E-04
ENSMUSG00000024843	Chka	7.819915	5.024614	5.94E-30	0.310079	-2.6843	9.03E-02	0.244882	-3.15174	1.75E-04	0.17992	-3.5424	2.65E-04	0.223904	-3.26309	1.66E-04	0.256525	-3.04734	4.64E-04
ENSMUSG00000029822	Osbpl3	2.21395	5.007585	2.91E-40	0.072572	-2.87037	1.26E-02	0.038263	-3.91248	1.43E-07	0.101493	-2.50003	8.44E-04	0.089562	-2.71528	7.41E-05	0.043479	-3.68973	4.03E-07
ENSMUSG00000003617	Cp	46.4828	4.998857	3.90E-15	2.51362	-2.2562	1.06E-02	1.35833	-3.27498	3.46E-09	1.134879	-3.48874	1.21E-08	1.343424	-3.27361	1.05E-09	1.260884	-3.34934	7.92E-10
ENSMUSG00000027690	Slc2a2	3.842253	4.920625	7.29E-50	0.20452	-2.24625	1.89E-02	0.153051	-2.7928	1.67E-06	0.04152	-4.54012	6.35E-10	0.1262	-3.05201	2.00E-07	0.12174	-3.08358	2.03E-07
ENSMUSG00000052562	Slc22a30	1.838188	4.900161	3.68E-31	0.158338	-1.51884	4.19E-01	0.045915	-3.40603	2.64E-05	0.02768	-3.98943	2.90E-05	0.065136	-2.90052	3.15E-04	0.039131	-3.569	2.02E-05
ENSMUSG00000020429	Igfbp1	2.48477	4.851906	2.62E-33	0.184728	-1.75316	2.49E-01	0.068873	-3.29569	1.62E-05	0.05536	-3.52974	5.09E-05	0.069207	-3.26665	2.29E-05	0.08261	-2.99716	9.57E-05
ENSMUSG00000027513	Pck1	34.1605	4.851252	3.90E-15	1.827487	-2.28728	1.79E-02	1.415725	-2.7734	1.61E-06	0.95496	-3.30534	4.45E-07	1.025887	-3.23222	2.57E-08	0.860879	-3.47164	4.39E-09
ENSMUSG00000024479	Mal2	1.056196	4.797863	5.84E-38	0.052779	-2.23018	4.73E-02	0.03061	-3.14476	6.79E-06	0.032293	-2.99478	1.01E-04	0.044781	-2.60834	1.17E-04	0.030435	-3.0961	1.22E-05
ENSMUSG00000015970	Chdh	1.093434	4.78058	2.24E-37	0.059377	-2.12737	6.83E-02	0.03061	-3.19797	6.49E-06	0.036907	-2.87391	1.93E-04	0.056994	-2.32787	6.05E-04	0.017391	-3.83921	4.45E-07
ENSMUSG00000053646	Plxnb1	1.029114	4.760509	1.65E-34	0.065974	-1.89837	1.29E-01	0.026784	-3.2852	1.06E-05	0.02768	-3.15803	1.28E-04	0.020355	-3.5999	2.83E-06	0.056522	-2.22933	1.66E-03
ENSMUSG00000022037	Clu	148.6901	4.752316	3.90E-15	7.395716	-2.41073	7.84E-03	4.844074	-3.14568	7.06E-08	4.553358	-3.18868	5.20E-07	4.763048	-3.15337	2.88E-08	6.687031	-2.63428	3.13E-06
ENSMUSG00000060961	Slc4a4	2.234262	4.745106	2.38E-43	0.125351	-2.16122	3.44E-02	0.065047	-3.23173	4.49E-07	0.050747	-3.50436	1.26E-06	0.061065	-3.29676	2.12E-07	0.126088	-2.24827	2.19E-04
ENSMUSG00000030236	Slco1b2	2.718352	4.718323	1.61E-36	0.20452	-1.75664	1.76E-01	0.114788	-2.71865	5.62E-05	0.0692	-3.37297	1.92E-05	0.101775	-2.8717	3.15E-05	0.052174	-3.77865	2.38E-07
ENSMUSG00000054889	Dsp	1.553827	4.701257	1.09E-38	0.098961	-1.95983	8.18E-02	0.061221	-2.78412	1.96E-05	0.02768	-3.76823	2.03E-06	0.089562	-2.22209	5.98E-04	0.026087	-3.86955	8.00E-08
ENSMUSG00000027792	Bche	1.597836	4.698395	1.53E-40	0.079169	-2.30943	2.80E-02	0.072699	-2.58327	3.55E-05	0.073813	-2.50195	3.47E-04	0.032568	-3.6566	7.60E-08	0.052174	-3.00127	4.31E-06
ENSMUSG00000059434	Gckr	1.543672	4.670122	4.42E-31	0.065974	-2.50886	5.71E-02	0.057394	-2.86874	1.26E-04	0.036907	-3.39449	9.97E-05	0.073278	-2.50345	9.98E-04	0.065218	-2.646	5.04E-04
