# Supplementary figures and images for: Single-Cell Transcriptomes Reveal Characteristic Features of Mouse Hepatocytes with Liver Cholestatic Injury
Source: Cells. 2019 Sep 11;8(9):1069. doi: 10.3390/cells8091069 (PMC6770527; doi:10.3390/cells8091069)

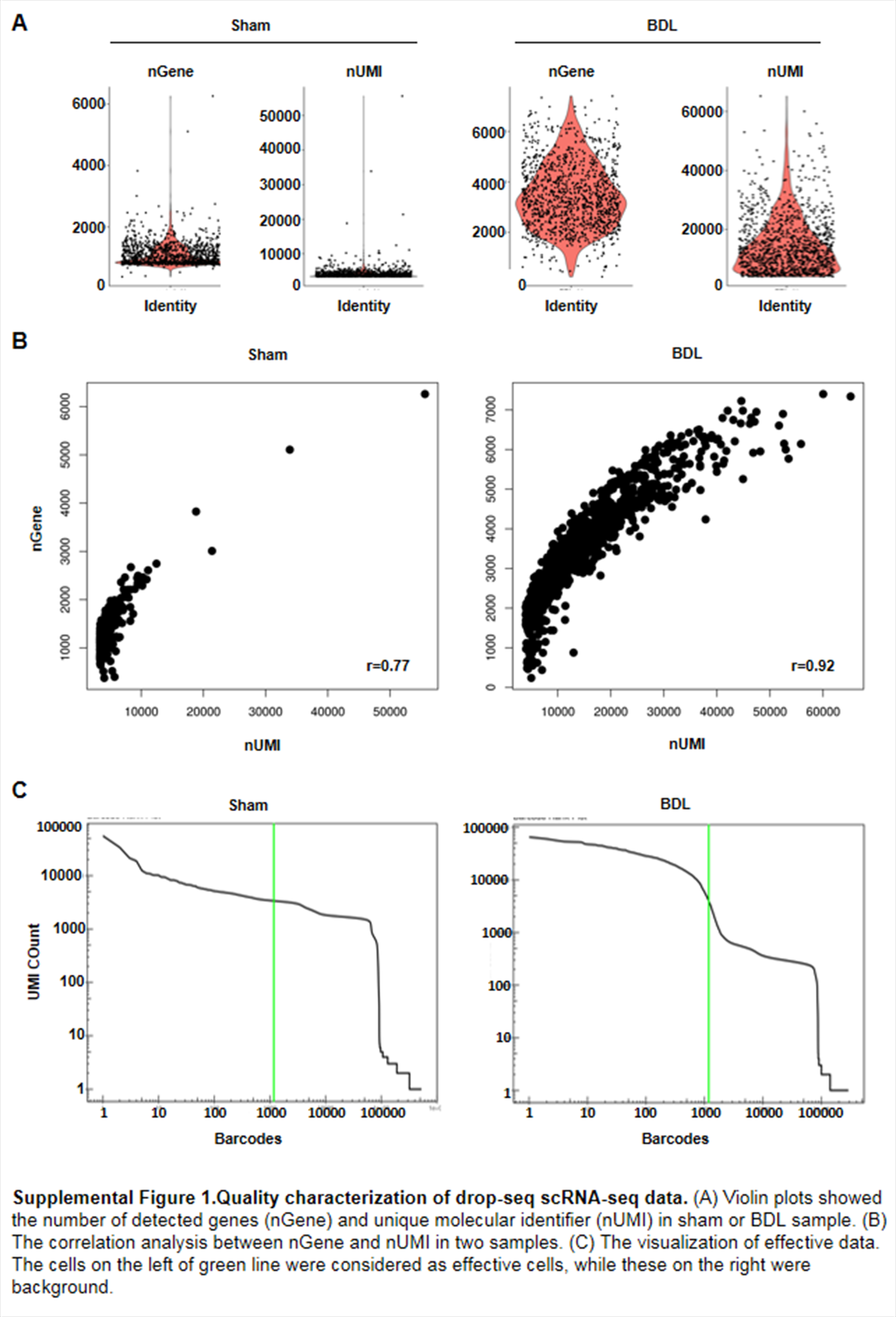

Supplement: Supplementary file 1 [file cells-08-01069-s001.zip › supplemental figures and tables/fig S1.tif]

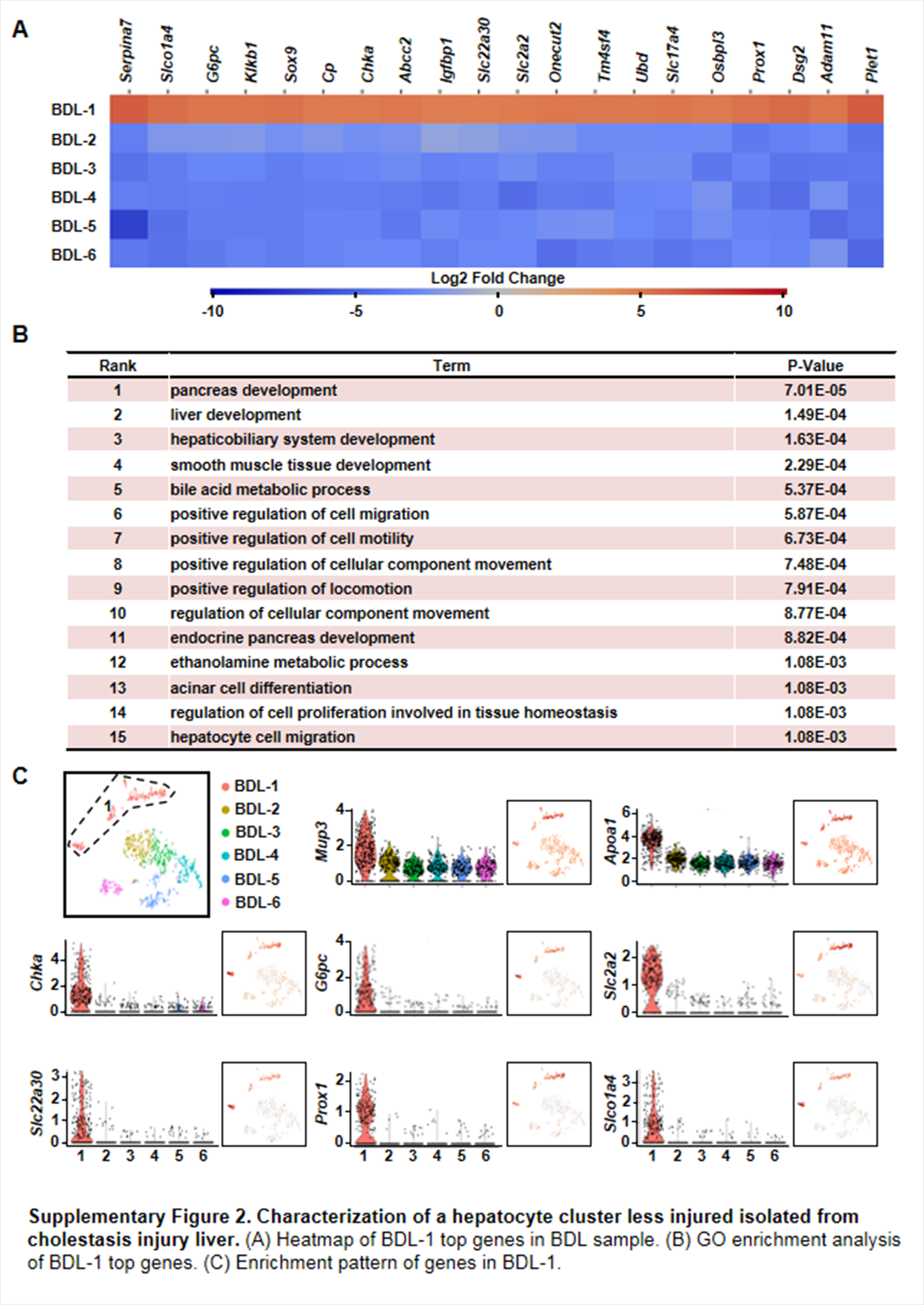

Supplement: Supplementary file 1 [file cells-08-01069-s001.zip › supplemental figures and tables/fig S2.tif]
